# Forward Genetic Dissection of Biofilm Development by Fusobacterium nucleatum: Novel Functions of Cell Division Proteins FtsX and EnvC

**DOI:** 10.1128/mBio.00360-18

**Published:** 2018-04-24

**Authors:** Chenggang Wu, Abu Amar Mohamed Al Mamun, Truc Thanh Luong, Bo Hu, Jianhua Gu, Ju Huck Lee, Melissa D’Amore, Asis Das, Hung Ton-That

**Affiliations:** a Department of Microbiology & Molecular Genetics, University of Texas McGovern Medical School, Houston, Texas, USA; b Houston Methodist Hospital Research Institute, Houston, Texas, USA; c Korean Collection for Type Cultures, Korea Research Institute of Bioscience and Biotechnology, Jeongeup-si, Jeollabuk-do, Republic of Korea; d Department of Molecular Biology and Biophysics, University of Connecticut Health Center, Farmington, Connecticut, USA; University of Alabama at Birmingham; Georgia Institute of Technology School of Biological Sciences

**Keywords:** EnvC, FtsX, *Fusobacterium nucleatum*, allelic exchange, biofilms, cell division, coaggregation, electron microscopy, filamentation, outer membrane

## Abstract

Fusobacterium nucleatum is a key member of the human oral biofilm. It is also implicated in preterm birth and colorectal cancer. To facilitate basic studies of fusobacterial virulence, we describe here a versatile transposon mutagenesis procedure and a pilot screen for mutants defective in biofilm formation. Out of 10 independent biofilm-defective mutants isolated, the affected genes included the homologs of the Escherichia coli cell division proteins FtsX and EnvC, the electron transport protein RnfA, and four proteins with unknown functions. Next, a facile new gene deletion method demonstrated that nonpolar, in-frame deletion of *ftsX* or *envC* produces viable bacteria that are highly filamentous due to defective cell division. Transmission electron and cryo-electron microscopy revealed that the Δ*ftsX* and Δ*envC* mutant cells remain joined with apparent constriction, and scanning electron microscopy (EM) uncovered a smooth cell surface without the microfolds present in wild-type cells. FtsX and EnvC proteins interact with each other as well as a common set of interacting partners, many with unknown function. Last, biofilm development is altered when cell division is blocked by MinC overproduction; however, unlike the phenotypes of Δ*ftsX* and Δ*envC* mutants, a weakly adherent biofilm is formed, and the wild-type rugged cell surface is maintained. Therefore, FtsX and EnvC may perform novel functions in *Fusobacterium* cell biology. This is the first report of an unbiased approach to uncover genetic determinants of fusobacterial biofilm development. It points to an intriguing link among cytokinesis, cell surface dynamics, and biofilm formation, whose molecular underpinnings remain to be elucidated.

## IMPORTANCE

Little is known about the virulence mechanisms and associated factors in F. nucleatum, due mainly to the lack of convenient genetic tools for this organism. We employed two efficient genetic strategies to identify F. nucleatum biofilm-defective mutants, revealing FtsX and EnvC among seven biofilm-associated factors. Electron microscopy established cell division defects of the Δ*ftsX* and Δ*envC* mutants, accompanied with a smooth cell surface, unlike the microfold, rugged appearance of wild-type bacteria. Proteomic studies demonstrated that FtsX and EnvC interact with each other as well as a set of common and unique interacting proteins, many with unknown functions. Importantly, blocking cell division by MinC overproduction led to formation of a weakly adherent biofilm, without alteration of the wild-type cell surface. Thus, this work links cell division and surface dynamics to biofilm development and lays a foundation for future genetic and biochemical investigations of basic cellular processes in this clinically significant pathogen.

## INTRODUCTION

The Gram-negative obligate anaerobe Fusobacterium nucleatum, described by Knorr in 1922 (1), has emerged as a key bridge-forming bacterium in the development of oral biofilms, owing to its ability to interact with many early and late colonizers of the oral cavity and tooth (2). Its prominence as a significant human pathogen has risen further since discoveries that *Fusobacterium* is also associated with preterm birth and colorectal cancer (CRC) (3–6). Recently, it has been shown that F. nucleatum mediates CRC chemoresistance by activation of the autophagy pathway (7). In spite of the versatile pathogenic potential of F. nucleatum, little is known about the mechanisms of fusobacterial virulence and associated factors, and virtually nothing is known about many other basic cellular processes in F. nucleatum, including protein transport, cell surface biogenesis, and cell division or cytokinesis. To date, less than a dozen of fusobacterial factors have been reported; these factors include FomA, FadA, Fap2, RadD, Aid1, FAD-I, and CmpA (6, 8–16), although more than 2,000 open reading frames (ORFs) are annotated in the genomes of many F. nucleatum strains (17–20) (see also https://www.patricbrc.org/). The majority of the aforementioned factors are adhesins, with Fap2, RadD, Aid1, and CmpA involved in polymicrobial interactions or coaggregation (10–12, 16); among these, only RadD and CmpA have been implicated in biofilm formation (10, 16).

A major impediment to basic research progress in *Fusobacterium* is the lack of convenient and robust genetic tools, though a few reverse and forward genetic analyses have been reported. The first double-crossover deletion mutant of the organism, the Δ*fadA* mutant, was generated by Han and colleagues more than a decade ago by a novel “sonoporation” method, whereby a suicide vector carrying a *fadA* deletion construct was delivered into F. nucleatum cells by ultrasound, permitting double homologous recombination events to replace the *fadA* gene with an erythromycin resistance cassette in a single step (14). How the sonoporation method promotes this one-step double-crossover allelic replacement still remains unknown, and Δ*fadA* and Δ*recA* mutants are among just a few deletion mutants that have been generated by this procedure thus far (21). More recently, a gene inactivation method via double crossover was described in which a chloramphenicol/thiamphenicol resistance cassette was inserted downstream of the *aid1* start codon, generating a truncated and out-of-frame *aid1* gene product, even though the *aid1* deletion construct was introduced into F. nucleatum via transformation (11). With regard to forward genetic approaches in F. nucleatum, Tn*5* transposon mutagenesis has been employed, producing a library of 1,200 Tn*5* clones subjected to a screen for mutants defective in hemagglutination with sheep red blood cells; this resulted in three hemagglutination-defective mutants, all mapped to *fap2* (12). To date, the genome coverage and randomness of this library have not been reported.

Considering the great clinical relevance of *Fusobacterium*, we have sought to improve the culture methods and the genetic and biochemical analyses of this fastidious obligate anaerobe colonizing the human oral cavity, gut, and uterine endometrium. Here, we report an improved Tn*5* transposon system that enabled us to generate a large number of Tn*5* mutants in F. nucleatum, roughly a collection of 24,000 independent isolates. We next performed a pilot screen for biofilm-defective mutants using a small set of 1,000 mutants from our library. Remarkably, the screen produced a total of 10 biofilm-defective mutants that we mapped to seven genes through molecular approaches. One class of mutants defined *ftsX* and *envC* as biofilm developmental factor genes, which encode as yet uncharacterized homologs of two known cell division factors of Escherichia coli FtsX and EnvC (22). Among other factors implicated in biofilm formation are genes predicted to code for an electron transport complex subunit, a C_4_-dicarboxylate transporter, a filamentous hemagglutinin, and an acetyltransferase, in addition to some hypothetical proteins. As a further improvement in the available tools for genetic analysis of *Fusobacterium*, we report the development of a facile allelic exchange method that allows the generation of unmarked, nonpolar, in-frame gene deletion mutants, which enabled the genetic and biochemical characterization of *ftsX* and *envC* in F. nucleatum. Both *ftsX* and *envC* deletion mutants are viable, which contrasts somewhat with the conditional essential phenotype of individual *ftsX* and *envC* deletion mutants in E. coli (22). The fusobacterial Δ*ftsX* and Δ*envC* mutants exhibit a severe cell morphological defect in that the mutant cells become highly filamentous due to a specific defect in cell separation as revealed by high-resolution electron microscopy (EM). In an effort to dissect the fusobacterial phenomenon further, we went on to carry out biochemical pulldown experiments using FtsX or EnvC as bait and identified a common set of proteins that interact with both FtsX and EnvC. We further show that the Δ*ftsX* and Δ*envC* mutants have a cell surface morphology defect that is separable from defective cell division. This specific defect in formation of a rugged surface possessed by the wild-type cells appears to be associated with the defect in biofilm development. Our study unveiled a hitherto unknown linkage between cytokinesis and cell envelope and biofilm formation, and we provide a genetic tool box for the community for future investigation into the mechanisms of fusobacterial virulence and fitness and its unique biology as a human commensal.

## RESULTS

### Novel biofilm-associated factors in Fusobacterium nucleatum uncovered by a forward genetic screen with a modified Tn*5* transposon.

Although a Tn*5* transposon system has previously been utilized for forward genetics in F. nucleatum (12), the efficiency of the system and the extent of genome coverage remain unknown. Consequently, with a goal to develop the system further, we modified the EZ-Tn5 system from Epicentre by replacing its kanamycin resistance cassette with the chloramphenicol/thiamphenicol resistance cassette present in the E. coli/F. nucleatum shuttle vector pHS30 (23). We used this EZ-Tn*5*-CT (CT stands for chloramphenicol/thiamphenicol) transposon in one transposome reaction, producing approximately 24,000 mutants, of which approximately a thousand clones were used in a pilot screen for biofilm-defective mutants. In this pilot screen, we seeded individual Tn*5* mutants into 96-well plates in the tryptic soy broth plus cysteine (TSPC) medium (see Materials and Methods) for biofilm formation at 37°C for 48 h inside an anaerobic chamber (see Fig. S1A in the supplemental material). Ten biofilm-defective mutants obtained were subsequently confirmed by a standard biofilm assay (24) in 12-well plates, as described in the legend to Fig. S1B.

10.1128/mBio.00360-18.1 FIG S1  A Tn*5* transposon screen identifies biofilm-associated factors in F. nucleatum. (A) Individual Tn*5* mutants were inoculated into 96-well plates for biofilm formation. Representative mutants defective in biofilm formation are circled in pink, whereas negative controls are circled in red. (B) Ten biofilm-defective Tn*5* mutants were further confirmed by a standard biofilm assay, whereby cells of WT and Tn*5* mutants were grown in a 12-well plate with 3 ml of TSPC in an anaerobic chamber at 37° for 48 h. Biofilms were washed and subsequently stained by 1% crystal violet. (C) Cells of WT and Tn*5* mutants in PBS were mixed in a sterile 96-well U-bottom plate with an equal volume (50 µl) of sheep red blood cells serially diluted from 2% (vol/vol). Hemagglutination was determined after 3 h of incubation at room temperature. (D) The *ftsX* loci of the *Fusobacterium* phylum and indicated bacteria are presented. The genes are color coded. Download FIG S1, PDF file, 2.5 MB. Copyright © 2018 Wu et al. 2018 Wu et al. This content is distributed under the terms of the Creative Commons Attribution 4.0 International license.

To identify the genes affected in these Tn*5* mutants, we adapted a mapping method termed single-primer one-step PCR (SOS-PCR) mapping, based on a published protocol (25), whereby one transposon-specific primer was used in a single PCR composed of three rounds of amplification (Fig. S2; primer P1). After SOS-PCR, the DNA products from the reactions were cleaned up and subjected to DNA sequencing reaction with another transposon-specific primer (Fig. S2; primer P2). The identities of the targeted genes were revealed by the chromosomal sequences adjoining the Tn*5* mosaic end (ME) motif in the sequenced amplicons, as summarized in Table 1. The Tn*1* and Tn*4* insertion mutations mapped to a gene homologous to that coding for the electron transport complex protein RnfA in Rhodobacter capsulatus (26, 27). Tn*2*/Tn*5* and Tn*3*/Tn*6* mutations mapped to a locus encoding homologs of the E. coli cell division protein FtsX and EnvC (22, 28), respectively. While the Tn*7* mutation targeted a gene of no known homolog or function, Tn*8* mutation affected a predicted transporter, Tn*9* mutation affected a filamentous hemagglutinin homolog, and Tn*10* mutation was located in a gene predicted to encode an *N*-acetyltransferase (Fig. S1B).

10.1128/mBio.00360-18.2 FIG S2  Single-primer one-step PCR (SOS-PCR) mapping of Tn*5* insertion sites. Tn*5* insertion sites were mapped by a one-step PCR, using a single primer (P1) targeting the *catP* gene of the Tn*5* transposon, with three stages of amplification employing alternate annealing temperatures. PCR products were purified and subjected to DNA sequencing using primer P2 that targets the mosaic end (ME) of Tn*5*. Download FIG S2, PDF file, 0.04 MB. Copyright © 2018 Wu et al. 2018 Wu et al. This content is distributed under the terms of the Creative Commons Attribution 4.0 International license.

**TABLE 1:** Mapping of biofilm-defective Tn*5* mutants

Tn*5* mutant	Target gene	Tn*5* location in the ORF (bp)^a^	Gene product
Tn*5*-*1*	HMPREF0397_1861	302 (585)	Electron transport complex subunit A
Tn*5*-*2*	HMPREF0397_1429	442 (927)	Cell division protein FtsX
Tn*5*-*3*	HMPREF0397_1428	1,141 (1,260)	Membrane-bound metallopeptidase, EnvC
Tn*5*-*4*	HMPREF0397_1861	300 (585)	Electron transport complex subunit A
Tn*5*-*5*	HMPREF0397_1428	571 (1,260)	Membrane-bound metallopeptidase, EnvC
Tn*5*-*6*	HMPREF0397_1429	807 (927)	Cell division protein FtsX
Tn*5*-*7*	HMPREF0397_0833	254 (525)	Hypothetical protein
Tn*5*-*8*	HMPREF0397_0437	60 (471)	C_4_-dicarboxylate transporter
Tn*5*-*9*	HMPREF0397_1811	3,032 (7,905)	Filamentous hemagglutinin
Tn*5*-*10*	HMPREF0397_1858	376 (450)	Acetyltransferase

a Numbers in parentheses indicate the gene length in base pair.

The fact that a hemagglutination-defective mutant affected biofilm development led us to test each of our mutants in a hemagglutination assay (see Materials and Methods), in which we measured the ability of the fusobacterial cells to bind and agglutinate sheep red blood cells. Figure S1C shows that while the Tn*9* mutant had a detectable hemagglutination defect, the Tn*1* and Tn*4* mutants each exhibited hemagglutination comparable to that of the wild-type strain. Intriguingly, the mutants with transposon insertions within *ftsX* and *envC* each showed elevated hemagglutination than the parental strain did (Fig. S1C).

**FIG 1:**
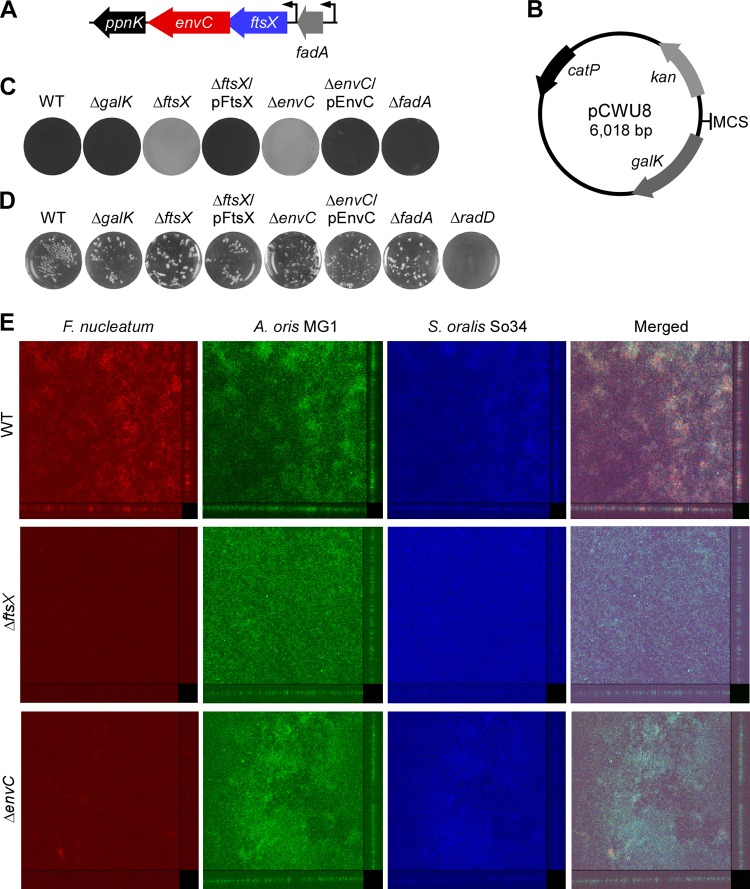
Roles of fusobacterial *ftsX* and *envC* in the development of monospecies and multispecies biofilms. (A) *ftsX* locus in the chromosome of F. nucleatum ATCC 23726, with *envC* adjacent to *ppnK*, which encodes an NAD+ kinase. Upstream of *ftsX* is *fadA* coding for the adhesin FadA (6); expression of genes in the *ftsX* locus and *fadA* appears to be controlled by individual promoters. (B) Map of the nonreplicative vector pCWU8 used to generate unmarked, in-frame gene deletion mutants in F. nucleatum. The kanamycin resistance cassette (*kan*), chloramphenicol/thiamphenicol resistance gene (*catP*), and *galK* are indicated. The multiple cloning sites (MCS) contain EcoRI, SacI, KpnI, BamHI, and SalI. (C) Fusobacterial strains were evaluated for their ability to form monospecies biofilm using crystal violet staining. (D) Interaction of fusobacteria and S. oralis So34 was determined by a standard coaggregation assay, with a *radD* mutant used as a negative control. (E) Three-species biofilms were analyzed by confocal laser scanning microscopy at a magnification of ×20, using 16S rRNA-oligonucleotide probes specific for F. nucleatum (red), A. oris (green), and S. oralis (blue); side and top views are presented. The results are representative of three independent experiments performed in triplicate.

We next focused on characterizing FtsX and EnvC mutants in this study due to several reasons. First, each of these genes was targeted by Tn*5* twice among the 10 mutants we isolated and that *ftsX* and *envC* are linked together in the genomes of several fusobacterial species whose genomes have been sequenced thus far, a gene arrangement different from well-characterized Gram-negative bacteria such as E. coli (Fig. S1D). It is noteworthy that our BLAST and homology searches revealed that the F. nucleatum FtsX protein sequence shared only 21.2% identity and 63.0% similarity with the E. coli FtsX protein (Fig. S3), whereas the fusobacterial EnvC protein sequence shared 25.1% identity and 66.9% similarity with the E. coli EnvC protein (Fig. S4). Overall, both fusobacterial proteins are more closely related to the clostridial proteins than to the E. coli counterparts (Fig. S5). We noted further that *ftsX*-*envC* genes are followed by *ppnK*, which is predicted to encode an NAD+ kinase (Fig. 1A), all apparently expressed as a single transcription unit (http://www.biocyc.org/). Second, we were also intrigued by the fact that the *ftsX* locus in *Fusobacterium* is devoid of a homolog of FtsE, an ATPase that is known to act in conjunction with FtsX in the septasome assembly and cytokinesis in E. coli and that forms a gene pair with *ftsX* in the E. coli genome (Fig. S1D).

10.1128/mBio.00360-18.3 FIG S3  Bacterial FtsX homologs. Shown is an alignment of amino acids of FtsX-like proteins. The GenBank accession codes are as follows: WP_005903510.1 (Fusobacterium nucleatum); WP_005974130.1 (Fusobacterium periodonticum); AAN82691.1 (Escherichia coli); WP_070142117.1 (Pseudomonas aeruginosa); WP_009968247.1 (Bacillus subtilis); CAA49620.1 (Mycobacterium tuberculosis); NP_721693.1 (Streptococcus mutans); CXG39040.1 (Streptococcus pneumoniae); WP_014318805.1 (Corynebacterium diphtheriae); WP_011068337.1 (Bifidobacterium longum); WP_011074182.1 (Shewanella oneidensis); WP_010963820.1 (Clostridium acetobutylicum); WP_011140650.1 (Gloeobacter violaceus); WP_005421374.1 (Vibrio fischeri). The multiple sequence alignment is generated using T-coffee (http://tcoffee.crg.cat/apps/tcoffee/do:regular). Download FIG S3, PDF file, 0.05 MB. Copyright © 2018 Wu et al. 2018 Wu et al. This content is distributed under the terms of the Creative Commons Attribution 4.0 International license.

10.1128/mBio.00360-18.4 FIG S4  Bacterial EnvC homologs. The sequences of EnvC-like proteins are shown aligned. The GenBank accession codes are as follows: EFG94960.1 (Fusobacterium nucleatum), WP_099960967.1 (Fusobacterium periodonticum), WP_061354207.1 (Escherichia coli), WP_043091244.1 (Pseudomonas aeruginosa), SGD49707.1 (Mycobacterium tuberculosis), CVP50706.1 (Streptococcus pneumoniae), WP_011070463.1 (Shewanella oneidensis), WP_049177113.1 (Clostridium botulinum), and WP_026029412.1 (Vibrio fischeri). The multiple-sequence alignment is generated using T-coffee (http://tcoffee.crg.cat/apps/tcoffee/do:regular). Download FIG S4, PDF file, 0.04 MB. Copyright © 2018 Wu et al. 2018 Wu et al. This content is distributed under the terms of the Creative Commons Attribution 4.0 International license.

10.1128/mBio.00360-18.5 FIG S5  Phylogenetic analysis of FtsX and EnvC homologs. (A and B) Phylogenetic trees of FtsX (A) and EnvC (B) homologs were reconstructed with the maximum likelihood method using the program Mega 6. The FtsX tree was rooted with lipoprotein transporter subunit LolE (FN0581) from F. nucleatum ATCC 23726, whereas the EnvC tree was unrooted. The GenBank accession numbers of these proteins are shown in Fig. S3 and S4. Download FIG S5, PDF file, 0.1 MB. Copyright © 2018 Wu et al. 2018 Wu et al. This content is distributed under the terms of the Creative Commons Attribution 4.0 International license.

### F. nucleatum mutants devoid of FtsX and EnvC cell division homologs are viable.

A consequence of transposon insertion mutagenesis is the undesirable polar effects affecting downstream gene expression, which can confound genetic analysis and interpretation. Therefore, to avoid potential polar effects, we desired to generate nonpolar, in-frame *ftsX* and *envC* deletion mutants. The limited availability of genetic tools for F. nucleatum led us first to develop an efficient and versatile gene deletion method suitable for this organism that is based on allele exchange mediated by homologous recombination. To that end, we took advantage of the inability of wild-type fusobacteria to grow in the presence of 2-deoxy-d-galactose (2-DG) (Fig. S6A and B), a compound that is metabolically converted by endogenous galactokinase (GalK) to toxic 2-deoxygalactose-1-phosphate in cells (29, 30). In contrast, a mutant strain lacking *galK* (Δ*galK* mutant) was able to grow in the medium supplemented with 0.25% 2-DG (as shown in Fig. S6B and C), while complementation of the strain with GalK expressed from the pCWU6 vector, which harbors a chloramphenicol resistance gene, *catP*, conferred 2-DG sensitivity (Fig. S6D). Thus, we were able to utilize GalK as a counterselectable marker in our allele exchange experiments, whereby a suicide vector pCWU8 (Fig. 1B), which carries *galK*, *catP*, and a kanamycin resistance gene (Fig. S6D), was employed in the three simple steps described below.

10.1128/mBio.00360-18.6 FIG S6  Development of a robust gene deletion method in F. nucleatum. (A to C) The wild-type strain, INT (integration of the suicide plasmid pCWU5 into the chromosome of the WT strain), Δ*galK* deletion mutant, and this mutant expressing GalK from a plasmid were grown on TSPC agar plates in the presence or absence of thiamphenicol or 2-deoxy-d-galactose (2-DG). (D) The E. coli/F. nucleatum shuttle vector pHS30 (23) was modified to include multiple cloning sites (MCSs) and mCherry under control of the C. diphtheriae *rpsJ* promoter, generating pCWU6. Separately, the *amp* gene of pUC19 was replaced by the *catP* gene of pHS30 (23) to generate pCWU5, which was used to clone the *galK* gene under control of the F. nucleatum *FN1529* promoter, generating the deletion vector pCWU8. (E) diagram of the Clostridium perfringens vector pCM-galK (44), which can be used in F. nucleatum. Download FIG S6, PDF file, 0.6 MB. Copyright © 2018 Wu et al. 2018 Wu et al. This content is distributed under the terms of the Creative Commons Attribution 4.0 International license.

For generation of individual *ftsX* and *envC* deletion mutants, 1-kb flanking DNA sequences from upstream and downstream of each gene, were cloned into pCWU8, and the resulting vectors were electroporated into the *galK* mutant of F. nucleatum. Integration of the recombinant vector into the bacterial chromosome was selected for by thiamphenicol, a chloramphenicol derivative. However, the integrant strains were unable to grow in the presence of 2-DG due to GalK expression from the integration vectors (Fig. S6B). Recombinants, in which excision of the vector sequence took place, leading to generation of wild-type or mutant alleles, were then selected on agar plates supplemented with 0.25% 2-DG. The presence of mutant alleles was screened for by PCR amplification of the targeted locus, and the loss of gene product was subsequently confirmed by Western blotting. While we succeeded in making the desired Δ*ftsX* and Δ*envC* mutants, several attempts to generate a similar deletion mutant of *ppnK*, the gene downstream of *envC*, were unsuccessful, suggesting that *ppnK* is an essential gene.

### FtsX and EnvC are required for biofilm formation.

The viable Δ*ftsX* and Δ*envC* mutants generated were subjected to the biofilm assay as mentioned above. In agreement with results obtained with the transposon insertion mutants, deletion of *ftsX* or *envC* abrogated biofilm formation compared to the parental strain and the *galK* mutant, and ectopic expression of each gene could rescue the biofilm defect (Fig. 1C). By comparison, deletion of *fadA* (14), the gene immediately upstream of *ftsX*, did not cause any noticeable biofilm defects (Fig. 1C, Δ*fadA*). Importantly, it is noteworthy that none of these mutants were defective in coaggregation with the Gram-positive early colonizer Streptococcus oralis (Fig. 1D), a cell-to-cell adherence process that precedes biofilm formation (31) and requires a coaggregation factor RadD in F. nucleatum (10).

We next investigated whether *ftsX* and *envC* are also required for formation of multispecies biofilms. To do this, we adapted an *in vitro* biofilm assay (32), whereby S. oralis (10^7^ CFU) cells grown overnight were washed and seeded onto 10% saliva-coated microwell dishes; after 2-h growth at 37°C, unattached cells were aspirated, and fresh media containing the Gram-positive early colonizer Actinomyces oris and F. nucleatum cells (10^8^ CFU each) were added. Subsequently, the presence of each bacterial species in the biofilms was monitored after 48-h growth in an anaerobic chamber using fluorescent DNA probes specific for each species (see Materials and Methods). As shown in Fig. 1E, wild-type fusobacteria were abundantly detected in the multispecies biofilm, whereas the Δ*ftsX* and Δ*envC* mutant strains were scarcely observed. Of note, the growth of F. nucleatum, A. oris, and S. oralis cells was not affected in the two biofilm conditions, i.e., parental and mutant fusobacteria. Altogether, the results support the role of FtsX and EnvC in the formation of monospecies as well as multispecies biofilms.

### FtsX and EnvC deletion mutations are defective in fusobacterial cytokinesis.

**FIG 2:**
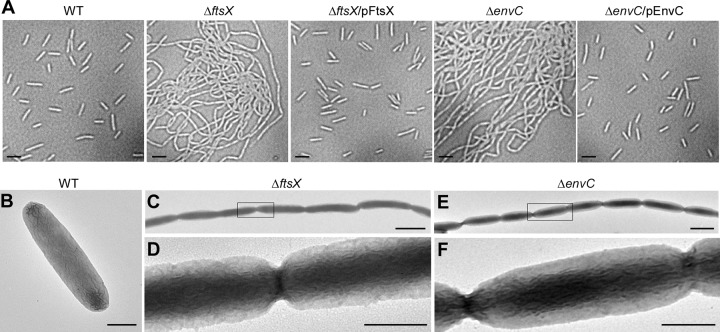
Microscopic studies of the *ftsX* and *envC* mutants. (A) Cell morphology of wild-type (WT) F. nucleatum and its isogenic derivatives was analyzed by phase-contrast microscopy. Bars, 2 µm. (B to F) Fusobacterial cells of wild-type (B), Δ*ftsX* (C and D), and Δ*envC* (E and F) strains were immobilized on carbon-coated nickel grids and stained with 1% uranyl acetate prior to viewing with a transmission electron microscope. Enlarged areas of panels C and E are shown in panels D and F. Bars, 0.5 µm.

**FIG 3:**
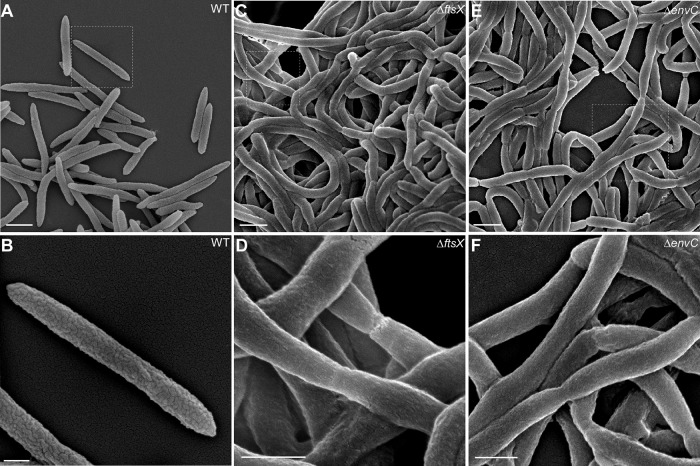
Altered cell surface of the *ftsX* and *envC* mutants revealed by scanning electron microscopy. (A to F) Exponential-phase F. nucleatum cells. Wild-type (A and B), Δ*ftsX* (C and D), and Δ*envC* (E and F) strains were immobilized on coverslips and fixed with 2.5% glutaraldehyde prior to viewing by a scanning electron microscope. Enlarged areas of panels A, C, and E are shown in panels B, D, and F, respectively. Bars, 1 µm (A, C, and E) and 0.5 µm (B, D, and F).

In E. coli, the loss of *ftsEX* or *envC* results in a minor defect in cell division under permissive conditions; the mutant cells appear to be slightly elongated (22). To evaluate whether this is the case in F. nucleatum, we examined Δ*ftsX* and Δ*envC* mutants by microscopy. By phase-contrast microscopy, both Δ*ftsX* and Δ*envC* fusobacterial mutants exhibited long filaments, in contrast to the small rods of the parental and complementing strains (Fig. 2A). To characterize the basis of filamentation, we immobilized fusobacterial cells on nickel grids, stained the fusobacteria with 1% uranyl acetate, and viewed them by transmission electron microscopy (TEM). As shown in Fig. 2C to F, the long chain of the *ftsX* and *envC* mutant fusobacteria was held together in chains with constricted but complete septa. This phenotype was also confirmed by cryo-electron microscopy, in which cells were quickly frozen in liquid ethane to preserve their native state (Fig. S7). Compared to the parental strain, which displayed normal septation leading to cell division (Fig. S7C), the Δ*ftsX* and Δ*envC* mutants still maintained complete septa displaying separated cytoplasm but lacking outer membrane invagination (Fig. S7B and D). Finally, the wild-type and mutant strains were further examined by scanning electron microscopy. Remarkably, compared to the wild-type fusobacterial cells, whose surface is rugged (Fig. 3A and B), the Δ*ftsX* and Δ*envC* chained cells displayed a very smooth surface (Fig. 3C to F). Together, these results demonstrate that both FtsX and EnvC are involved in cell division in fusobacteria and that in the absence of *ftsX* or *envC*, the cell envelope is visibly altered, lacking the microfolds that characterize the wild-type cells.

10.1128/mBio.00360-18.7 FIG S7  Cryo-electron microscopic analysis of the Δ*ftsX* and Δ*envC* mutants. (A and B) The mutant cells were grown in TSPC to exponential phase and analyzed by cryo-EM. A montage in panel A was constructed from a series of images of a filamentous cell. Bar, 2 µm (A). A highlighted area in panel A is enlarged in panel B. Bar, 0.1 µm (B). (C and D) Cells of the WT and Δ*envC* strains were analyzed by cryo-electron tomography. Arrows indicate the site of septal constriction. Bars, 0.1 µm. Download FIG S7, PDF file, 1.8 MB. Copyright © 2018 Wu et al. 2018 Wu et al. This content is distributed under the terms of the Creative Commons Attribution 4.0 International license.

**FIG 4:**
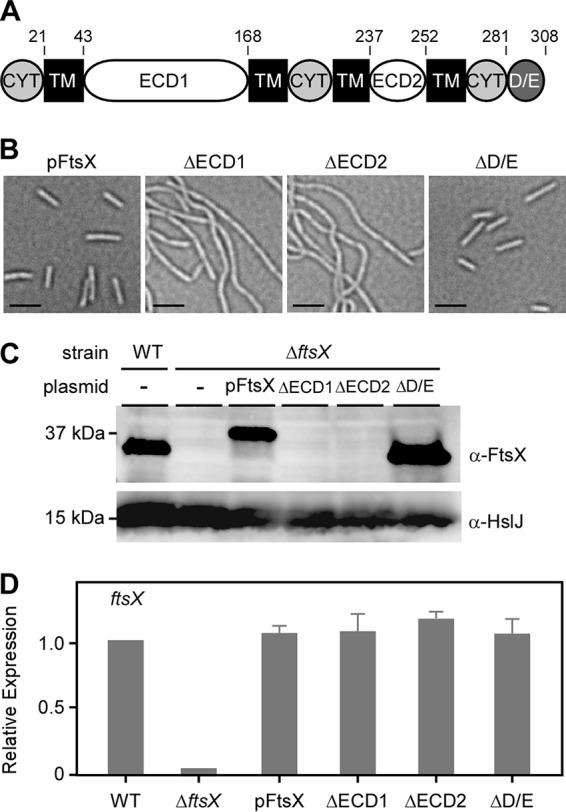
Requirement of the extracellular domains ECD1 and ECD2 for FtsX functionality. (A) Predicted structural domains of FtsX, showing four transmembrane (TM) domains, two extracellular loops (ECD1 and ECD2), three cytoplasmic regions (CYT), and a unique C-terminal aspartic acid/glutamic acid-rich domain (D/E). Positions of amino acids are indicated above the map. (B) Phase-contrast microscopic images of the complementing *ftsX* strain and its derivatives lacking the ECD1, ECD2, or D/E-rich domain. Bars, 2 μm. (C) Whole-cell lysates of fusobacterial strains grown to mid-log phase and normalized by optical density were analyzed by immunoblotting with antibodies against FtsX (anti-FtsX [α-FtsX]) and HslJ (anti-HslJ [α-HslJ]); the latter serves as a loading control. The positions of molecular mass markers (in kilodaltons) are indicated to the left of the blot. Of note, pFtsX contains His and Flag tags. (D) The mRNA expression level of *ftsX* in indicated strains was determined by quantitative reverse transcription-PCR (qRT-PCR) relative to the parental strain. Values are averages plus standard deviations (error bars) from two independent experiments performed in triplicate.

By domain analysis using TMHMM, Phobius, and CCTOP programs (33–35), F. nucleatum FtsX was predicted to have four transmembrane (TM) domains with three cytoplasmic regions and two extracellular domains (ECD) similar to the E. coli counterpart; F. nucleatum FtsX also contained a C-terminal aspartic acid/glutamic acid-rich (D/E) domain, apparently present only in oral fusobacterial FtsX-like proteins (Fig. 4A). To assess whether the presumed functions of FtsX in the cytoplasm and periplasm are conserved in fusobacterium, we generated FtsX mutants that lack parts of extracellular domain 1 (ECD1) (missing residues 61 to 141), ECD2 (missing residues 241 to 249), or D/E domain; note that ECD1 is flanked by two TM domains (residues 21 to 43 and residues 168 to 190), whereas ECD2 is flanked by the other two TM domains (residues 210 to 237 and residues 252 to 269). Plasmids carrying these constructs were individually introduced into the Δ*ftsX* mutant. Compared to the wild-type FtsX, deletion of ECD1 or ECD2 resulted in the same filamentous phenotype as that of deletion of *ftsX*, whereas the lack of the D/E domain did not alter the cell morphology compared to the wild-type FtsX (Fig. 4B), suggesting that the D/E motif is functionally dispensable.

To assess the defect of the ECD domain deletions further, we analyzed the whole-cell lysates of these strains by immunoblotting, utilizing polyclonal antibodies generated against FtsX, while FtsX protein was abundantly present in the mutant lacking the D/E domain, no signal was detected in strains expressing FtsX mutants lacking parts of ECD1 or ECD2 (Fig. 4C), though there was no defect in transcription of the mutant genes as determined by quantitative reverse transcription-PCR (qRT-PCR) (Fig. 4D). Considering that the deletion mutants were designed so as not to disturb the transmembrane topology, the results suggest that the ECD1 and ECD2 domains might be involved in membrane insertion of FtsX or that the mutations have affected the folding and stability of FtsX.

### Identification of proteins that interact with FtsX and EnvC.

The well-studied FtsX protein of E. coli interacts with its partner EnvC in the periplasm, which activates an amidase required for cell wall hydrolysis and cell separation during cell division (22). E. coli FtsX also forms a complex with FtsE in the cytoplasm and interacts with the divisome membrane anchor protein FtsA, thus orchestrating the progression of divisome assembly further and stimulating the ultimate cell separation event (36).

**FIG 5:**
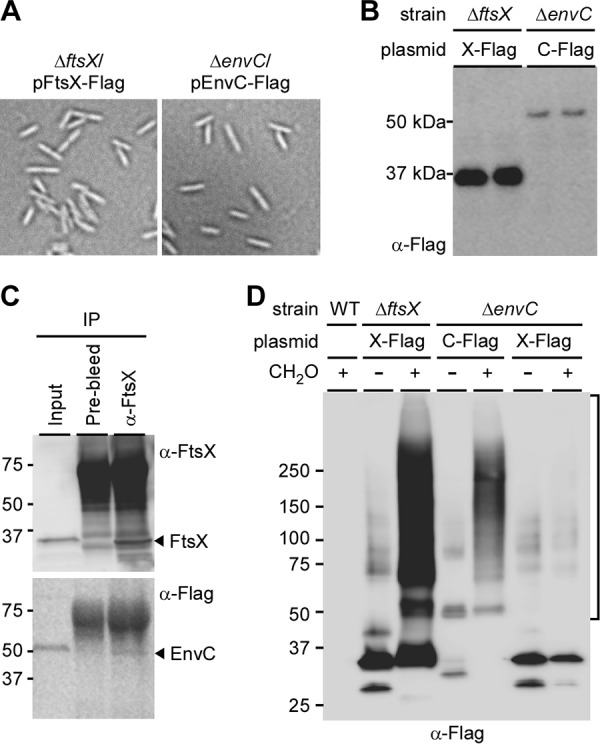
Interactions of FtsX and EnvC. (A) Functionality of a plasmid expressing Flag-tagged FtsX (X-FLAG) or EnvC (C-FLAG) in the Δ*ftsX* or Δ*envC* mutant was examined by phase-contrast microcopy. (B) Cell lysates of both strains in panel A were subjected to immunoblotting with antibodies against the Flag tag (anti-Flag [α-Flag]) with duplicate samples. (C) Cell membrane preparations from the Δ*envC* mutant expressing C-FLAG were subjected to coimmunoprecipitation using IgG control (prebleed) or anti-FtsX antibodies. Protein samples derived from the input (before immunoprecipitation [IP]), IgG^−^, and anti-FtsX fractions were immunoblotted with anti-FtsX and anti-Flag antibodies. (D) Cells of the indicated strains were treated with formaldehyde (CH_2_O) or mock treated. Protein samples were analyzed by Western blotting with α-Flag antibody.

To identify the interacting partners of FtsX and EnvC in *Fusobacterium*, we constructed dual His/Flag-tagged FtsX and EnvC. The mutant cells expressing these recombinant proteins were no longer filamentous, thus demonstrating intact functionality of these proteins (Fig. 5A). Western blotting analysis with Flag-tagged antibodies detected these proteins in the cells, though EnvC signal was visibly lower than that of FtsX (Fig. 5B).

To determine whether FtsX and EnvC interact in F. nucleatum, we performed a coimmunoprecipitation assay, whereby FtsX from the membrane lysates of the Δ*envC* mutant expressing His/Flag-tagged EnvC was captured by polyclonal antibodies against recombinant FtsX, followed by treatment with protein A beads. Bound proteins were then analyzed by immunoblotting with anti-FtsX and anti-Flag antibodies. As expected, anti-FtsX antibodies were able to capture EnvC along with FtsX, whereas no FtsX and EnvC signals were detected in the control samples using prebleed sera (Fig. 5C).

We next sought to identify other potential interactors of FtsX and EnvC present in fusobacteria. To trap weak interactions, the Δ*ftsX* and Δ*envC* mutant cells expressing dual His/Flag-tagged proteins were first subjected to formaldehyde (FA) cross-linking prior to membrane lysis. His/Flag-tagged FtsX and EnvC proteins were then purified from the membrane lysates by tandem affinity purification, and protein samples were analyzed by sodium dodecyl sulfate-polyacrylamide gel electrophoresis (SDS-PAGE) in duplicate (see Materials and Methods). With Western blotting analysis using monoclonal antibodies against the Flag tag, no signal was observed in the parental strain upon FA treatment as expected (Fig. 5D, WT [wild-type] lane). In contrast, the Δ*ftsX* mutant expressing His/Flag-tagged FtsX produced four major bands ranging from 25 to 100 kDa that were detected in samples without cross-linking, and a smear pattern of bands ranging from 25 kDa to greater than 250 kDa in the FA-treated sample (Fig. 5D, compare the two Δ*ftsX* lanes with and without FA). The Δ*envC* mutant expressing His/Flag-tagged EnvC gave rise to fewer bands in the samples without FA, with bands migrating around the 50- and 75-kDa markers (Fig. 5D, Δ*envC* lane without FA); these bands may correspond to EnvC monomers and FtsX-EnvC heterodimers, as the theoretical molecular masses of the FtsX and EnvC monomers are 35.5 kDa and 47.9 kDa, respectively. FA treatment of the Δ*envC* mutant expressing His/Flag-tagged EnvC produced a smear pattern of bands ranging from 50 kDa to greater than 250 kDa (Fig. 5D, Δ*envC* lane with FA). Furthermore, tandem affinity purification of His/Flag-tagged FtsX from the Δ*envC* mutant with or without FA resulted in a banding pattern similar to that seen in the samples of the Δ*ftsX* mutant expressing His/Flag-tagged FtsX without cross-linking (Fig. 5D, Δ*envC* lanes with and without FA). Clearly, multiple proteins interact with both FtsX and EnvC in *Fusobacterium*.

To identify these interacting proteins, gel pieces containing high-molecular-mass species of 50 kDa and greater (as shown in Fig. 5D) were excised and analyzed by tandem mass spectrometry. Proteins identified from samples of the WT lane and Δ*ftsX* lane without FA were used as the background control for proteins identified in the Δ*ftsX* lane with FA (Fig. 5D); likewise, proteins identified from samples of the WT lane and Δ*ftsX* lane with FA served as background for the analysis of proteins identified in Δ*envC* lane with FA (Fig. 5D). Results of mass spectrometry revealed that a similar number of proteins copurified with His/Flag-tagged FtsX and EnvC (Table S1), 16 of which were found in both FtsX and EnvC samples (Table 2). The common interacting proteins include FtsX, EnvC, a murein hydrolase activator homologous to E. coli NlpD originally proposed to be involved in cell wall formation (37), tetratricopeptide repeat (TPR) and MORN family proteins, as well as a significant number of proteins with no known functions. Intriguingly, an *N*-acetylmuramoyl-l-alanine amidase termed AmiD3 was copurified with EnvC (Table S1) and FtsX. We made several systematic attempts to delete *nlpD* and *amiD3* genes, but we were unsuccessful in obtaining the desired deletion mutants, indicating that these cell division proteins are essential in F. nucleatum. Notably, several attempts to construct a Δ*ftsX*-Δ*envC* double mutant have also failed, suggesting that this genetic combination is synthetic lethal. Further characterization of the functions of FtsX and EnvC and their interacting proteins in cell division and biofilm development will benefit from a conditional gene knockout system for F. nucleatum, which is currently under development in our laboratory.

10.1128/mBio.00360-18.8 TABLE S1 FtsX-associated proteins (A) and  EnvC-associated proteins (B) identified by mass spectrometry. Download TABLE S1, PDF file, 0.04 MB. Copyright © 2018 Wu et al. 2018 Wu et al. This content is distributed under the terms of the Creative Commons Attribution 4.0 International license.

**TABLE 2:** Common proteins copurified with Flag-tagged FtsX and EnvC

Protein no.^a^	Gene ID^b^	Predicted function
1	HMPREF0397_1429	Cell division protein FtsX
2	HMPREF0397_1428	Membrane-bound metallopeptidase, EnvC
3	HMPREF0397_0565	Peptidase, M23 family; NlpD
4	HMPREF0397_1662	SPFH domain/band 7 family protein
5	HMPREF0397_1026	TPR family protein
6	HMPREF0397_1393	MORN variant protein
7	HMPREF0397_0968	TPR family protein
8	HMPREF0397_1313	MORN repeat protein; YwqK
9	HMPREF0397_0259	Alpha/beta hydrolase
10	HMPREF0397_1251	GDYXXLXY protein
11	HMPREF0397_1477	Hypothetical protein
12	HMPREF0397_0789	Hypothetical protein
13	HMPREF0397_0605	Hypothetical protein
14	HMPREF0397_0711	Hypothetical protein
15	HMPREF0397_2044	Hypothetical protein
16	HMPREF0397_0561	Hypothetical protein

a Numbers given to the 16 proteins found in both FtsX and EnvC samples.

b Gene ID, gene identifiers.

### Novel functions of FtsX and EnvC in fusobacterial cell surface morphogenesis and biofilm development.

**FIG 6:**
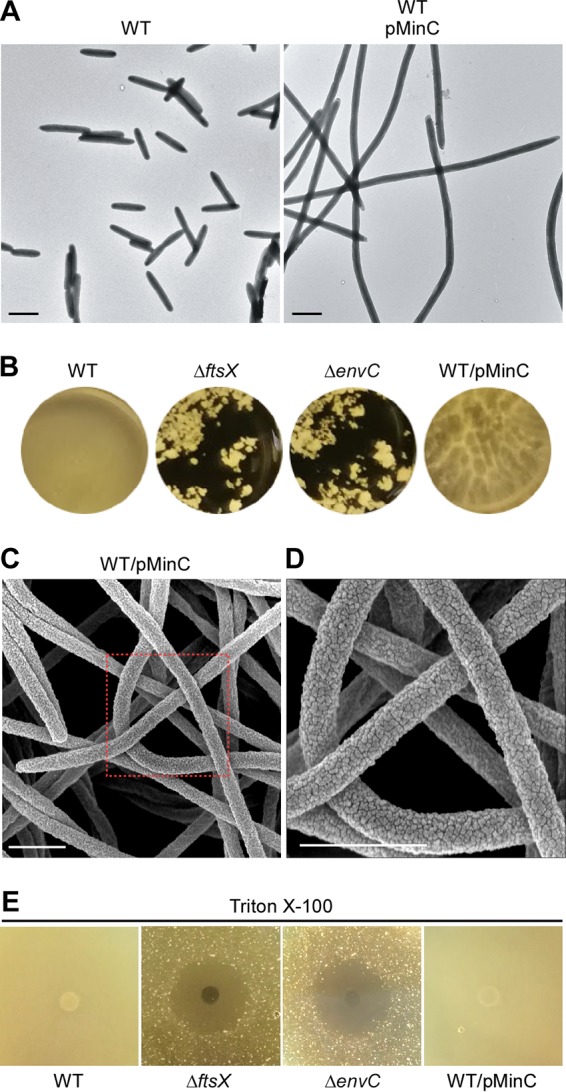
Characterization of MinC-overproducing cells. (A) Cells of the wild-type strain and this strain that overexpressed fusobacterial MinC were subjected to electron microscopy with negative staining as described in the legend to Fig. 2. Bars, 1 µm. (B) Biofilms of the indicated strains were cultivated in multiwell plates and imaged. (C and D) Cells of the wild-type strain overexpressing MinC were subjected to scanning electron microscopy as described in the legend to Fig. 3. An enlarged area in panel C is shown in panel D. Bars, 1 µm. (E) Individual wells on agar plates were placed with 50 microliters of 20% Triton X-100, and wells containing diffuse detergent were overlaid with culture aliquots of indicated strains. Plates were incubated at 37°C in an anaerobic chamber for 72 h prior to imaging. The results are representative of three independent experiments.

Although the Δ*ftsX* and Δ*envC* mutants fail to form biofilms on their own, they incorporate themselves in the biofilm formed by the wild-type organism (data not shown). To further establish a causal link between the blocked cell division and the altered surface morphology to the defect in biofilm formation, we tested whether biofilm development is affected by blocking cell division by another means whereby FtsX and EnvC are kept intact. For this purpose, we took advantage of the well-known bacterial cell division inhibitor MinC, a homolog of which is encoded in F. nucleatum. When F. nucleatum MinC was overproduced constitutively, cell division was blocked efficiently without affecting cell viability, similar to the phenotype of the *ftsX* or *envC* mutant (compare Fig. 6A with Fig. 2A). In contrast to the cell clumping and a pronounced defect in biofilm formation displayed by the *ftsX* or *envC* mutant, however, the MinC-induced cell filamentation only mildly affected biofilm development. First, the biofilm was visibly not as uniform as that produced by the wild type (Fig. 6B). Second, the biofilm was sensitive to washing by salt solutions unlike the wild type (Fig. 6B). Electron microscopy showed that the long filaments produced by MinC overproduction lacked the constrictions that are clearly visible in the filaments of the Δ*ftsX* or Δ*envC* mutant (compare Fig. 6C with Fig. 3D to F). Most importantly, scanning EM revealed that the MinC-induced filaments still possessed a rugged cell surface that is identical to that seen with wild-type cells, with a surface covered by microfolds very similar in size, shape, and density to those of the wild type (compare Fig. 6D with Fig. 3B). The absence of microfolds in the Δ*ftsX* or Δ*envC* mutant suggested that the outer membrane in these mutant cells might be compromised. To test this, we examined whether the two mutants are affected by treatment with Triton X-100, a nonionic detergent that solubilizes the bacterial inner membranes, but not the outer membranes (38), using a plating assay (see Materials and Methods). Indeed, compared to the parental and MinC-overexpressing strains, the two mutants were highly sensitive to the detergent, which was evident from the large zones of inhibition (Fig. 6E). The results suggest that the failure of the Δ*ftsX* or Δ*envC* mutant to form biofilms might be due to their altered outer membrane architecture *per se* and not by their filamentation. We conclude that FtsX and EnvC play some novel roles in the outer membrane biogenesis in *Fusobacterium* that is also critical for biofilm formation in a fashion independent of cell division.

## DISCUSSION

F. nucleatum is a notorious human pathogen that is uniquely associated with multiple and distinct human pathologies, from playing a coveted role in orchestrating the development of the most complex microbial biofilm of the human body to enhancing tumorigenesis in the colon and colonizing the uterine cavity and potentially the fetus to induce preterm birth. However, to date, our knowledge of the virulence factors of this pathogen is quite limited, which might be rationalized by the fact that *Fusobacterium* is a fastidious organism and lacks facile genetic manipulation systems that can provide the power and convenience needed for dissecting the host pathogen interactions that underlie microbial pathogenesis. This technical gap might stem from a strong genetic restriction barrier present in certain strains of F. nucleatum and the lack of counterselection methods for carrying out allelic exchange by homologous recombination. To that end, we report here a vastly improved transposon mutagenesis procedure for forward genetic screens in F. nucleatum (see Materials and Methods), leading to the identification of many uncharacterized biofilm-associated proteins of the organism for the first time (Fig. 1 and Table 1). We further report the development of an unmarked gene deletion method that permits a robust selection and generation of mutants undergoing double-crossover homologous recombination (see Fig. S6 in the supplemental material). Using this technology, we were able to generate rapidly and conveniently the nonpolar, in-frame gene deletion mutants of the newly identified biofilm factors, including FtsX and EnvC, two cell division proteins that were first identified in E. coli and that have been well characterized in E. coli (28).

In agreement with what has been known from studies of E. coli, we present experiments to demonstrate that FtsX and EnvC are involved in cell division in F. nucleatum. First, deletion of the *ftsX* or *envC* gene does not affect bacterial viability but results in cell filamentation readily observed by phase-contrast microscopy (Fig. 2). Second, by electron microscopy (Fig. 2 and Fig. S7), we show that the *ftsX* or *envC* mutant cells are joined at the septa, with membrane constriction clearly evident, indicating that FtsX and EnvC are not required for membrane constriction but that each plays a significant role in cell separation. F. nucleatum FtsX is predicted to harbor several conserved domains found in Streptococcus pneumoniae and E. coli FtsX proteins (22, 39), including two extracellular domains (ECD1 and ECD2). By deletion analysis, ECD1 and ECD2 appeared to be essential for FtsX stability, whereas a D/E motif, present only in the phylum *Fusobacterium*, appears to be dispensable (Fig. 4). It is not clear at this time why this motif is conserved in these organisms.

We went on to demonstrate that FtsX interacts with EnvC physically, as expected (Fig. 5C), and made an effort to identify additional interacting partners of each of these proteins, using recombinant proteins with a dual tag to perform pulldown assays and to isolate the trapped interactors by tandem affinity purifications. We identified a large number of common and novel interacting partners, many of which are hypothetical (Table 2). Intriguingly, one of these proteins belongs to the M23 family of peptidases (Table 2, HMPREF0397_0565); it contains conserved domains, including the peptidoglycan binding LysM and murein-DD-endopeptidase MepM domains, found in the E. coli lipoprotein NlpD (37). In E. coli, cell wall hydrolases are critical for regulated cleavage of cell wall materials connecting two dividing cells to facilitate cell separation, and NlpD was shown to activate the amidase AmiC, whereas EnvC activates AmiA and AmiB (40). Thus, it is likely that FtsX, EnvC, and HMPREF0397_0565 are part of the divisome in F. nucleatum. According to the model proposed by Yang and colleagues (22), conformational changes in the FtsEX complex, induced by ATP hydrolysis mediated by the ATPase FtsE, are conveyed to EnvC, which in turn activates enzymatic activities of the amidases. Our failed attempts to make a deletion mutant for the suspected NlpD homolog suggest that it is essential for F. nucleatum, which in itself is not a novel result but is also important due to its potential as a unique target for therapeutic development.

The identification of a putative amidase activator as an interactor of FtsX/EnvC leads to two logical questions. First of all, does the FtsX-EnvC-NlpD complex activate an amidase in F. nucleatum and if so, which amidase? Our pulldown assay, using EnvC as the bait, identified AmiD3 (HMPREF0397_0675) as one of the interacting partners (Table S1). If this predicted AmiD3 homolog is the expected amidase, genetic disruption of this gene should phenocopy the *ftsX* or *envC* mutant. Unfortunately, our failed attempts to make the deletion mutant suggest that this *amiD3* homolog is also essential, as is NlpD. A definitive test of the prediction must wait until we have successfully developed a conditional gene deletion system for F. nucleatum. Second, unlike E. coli and many other Gram-negative bacteria, an obvious *ftsE* homolog is not located adjacent to the *ftsX* locus in F. nucleatum (Fig. S1). Interestingly, however, the pulldown assay using FtsX as the bait identified HMPREF0397_1681, a predicted cation ABC superfamily ATP binding protein. It is tempting to postulate that HMPREF0397_1681 functions as FtsE in F. nucleatum. Further genetic and biochemical work will be needed to fully characterize the FtsEX-EnvC-amidase component of the F. nucleatum cell division apparatus. In this context, the surprising fact that our proteomic study did not yield FtsA as one of the interactors of FtsX (36) leaves a critical gap in this picture, and hence provides further impetus for a more directed set of studies to illuminate the process of cell division in *Fusobacterium*.

Perhaps the most intriguing puzzle that has emerged from our work is the unexpected and interconnected links among cell filamentation, surface morphology, and biofilm development. We showed that MinC overproduction leads to the formation of filaments, but it does not affect biofilm formation in the same way that is so clearly evident with the filaments produced by either *ftsX* or *envC* mutants. The MinC-induced filaments differ from the *ftsX* or *envC* filaments in two morphological respects. First, constriction is absent in MinC-induced filaments in agreement with what is known in E. coli. Second, the MinC-induced filaments of F. nucleatum maintain the same rugged cell surface morphology as the wild type that is completely absent in the filaments of *ftsX* or *envC* mutants. This remarkable deficiency in proper envelope morphogenesis in *ftsX* and *envC* mutants, their sensitivity to detergents, and the fact that a large body of functionally unknown proteins interact with FtsX and EnvC lead us to posit that this network of proteins participate in a cell surface morphogenetic pathway that is a major factor in the development of biofilm by *Fusobacterium*.

It is noteworthy that the *ftsX* or *envC* mutants are capable of mediating several distinct cell-cell interaction processes that we have examined. This includes coaggregation with streptococci, a process that requires the outer membrane adhesin RadD (10). Furthermore, the *ftsX* and *envC* mutants remain adhesive to red blood cells, and this hemagglutination appears to involve HMPREF0397_1811, predicted to encode a filamentous hemagglutinin (Fig. S1). The identification of additional biofilm-associated factors such as the electron transport complex subunit A, C_4_-dicarboxylate transporter, and acetyltransferase (Table 1) suggests that cell metabolism may play an important role in fusobacterial biofilm formation. Future efforts focusing on characterizing these factors utilizing the new genetic tools reported here promise to provide important further insights not only for biofilm formation but also for some of the fundamental processes of fusobacterial cell biology.

Notably, during the final stage of preparation of this article, a major publication appeared that described mouse xenograft studies and provided several compelling pieces of evidence for colon tumor’s dependence on fusobacteria and the sensitivity of the tumors to antibiotic therapy (41). The comprehensive transposon library we generated should facilitate the identification of new genetic elements potentially involved in the addiction of colon cancer to *Fusobacterium*.

## MATERIALS AND METHODS

### Bacterial strains, primers, plasmids, and media.

Bacterial strains and plasmids used in this study are listed in Table S2 in the supplemental material. F. nucleatum strains were grown in tryptic soy broth (TSB) supplemented with 1% Bacto peptone plus 0.25% freshly made cysteine (TSPC) or on TSPC agar plates with 1% vitamin K1-hemin solution or BBL Columbia agar with 5% sheep blood in an anaerobic chamber (5% CO_2_, 2% H_2_, and 93% N_2_). Streptococci were grown in brain heart infusion (BHI) supplemented with 0.5% glucose. A. oris was grown in heart infusion broth (HIB). E. coli strains were grown in Luria broth (LB). When needed, kanamycin, chloramphenicol, or thiamphenicol was added to the medium at a concentration of 50, 15, or 5 µg ml^−1^, respectively. Reagents were purchased from Sigma unless indicated otherwise.

10.1128/mBio.00360-18.9 TABLE S2  Bacterial strains and plasmids used in this study. Download TABLE S2, PDF file, 0.1 MB. Copyright © 2018 Wu et al. 2018 Wu et al. This content is distributed under the terms of the Creative Commons Attribution 4.0 International license.

### Plasmid construction. (i) pCWU5.

Primers catP-HS30-F (F stands for forward) and catP-HS30-R (R stands for reverse) (Table S3), each of which contained an AcII site for cloning purposes, were used along with pHS30 (23) as a template to PCR amplify a DNA segment encompassing the promoter, 5′ untranslated region (UTR), and coding sequence of the chloramphenicol/thiamphenicol resistance *catP* gene. The PCR product was digested with AcII and ligated into pUC19 precut with AcII to generate pCWU5.

10.1128/mBio.00360-18.10 TABLE S3  Primers used in this study. Download TABLE S3, PDF file, 0.1 MB. Copyright © 2018 Wu et al. 2018 Wu et al. This content is distributed under the terms of the Creative Commons Attribution 4.0 International license.

### (ii) pCWU5-ΔgalK.

Two-kilobase fragments upstream and downstream of *galK* were amplified from the genomic DNA of F. nucleatum strain ATCC 23726 using the primer sets galKupF/galkupR and galkdnF/galKdnR, respectively (Table S3). The fragments generated were digested with EcoRI/KpnI and KpnI/XbaI, respectively, and ligated into the vector pCWU5 precut with EcoRI/XbaI. The generated plasmid pCWU5-ΔgalK was confirmed by colony PCR and restriction enzyme digestion.

### (iii) pCWU7 and pCWU8.

Primer sets P1529-F/R and galK-F/R (Table S3) were used in the PCR amplification of a DNA fragment encompassing the 5′ promoter sequence and UTR of the highly expressed gene FN1529 (42) and the *galK* coding sequence, respectively, from the genomic DNA of F. nucleatum ATCC 25586 as the template, while appending SalI and NheI or NheI and HindIII sites to the DNA fragments, respectively. The amplified fragments were digested with SalI/NheI or NheI/HindIII, respectively, and ligated into the vector pCWU5 precut with SalI and HindIII, resulting in pCWU7, which was confirmed by restriction enzyme digestion and DNA sequencing. To generate pCWU8, the kanamycin resistance gene cassette (*kan*) from pJRD215 (24) was cloned into pCWU7 in its NdeI site.

### (iv) pCWU6.

The E. coli/F. nucleatum shuttle vector pHS30 (23) has only HindIII and SacI sites available for cloning. To expand its multiple cloning sites, the pHS30 vector was modified as follows. First, primers PrpsJcory-F and PrpsJcory-R (Table S3) were used to PCR amplify the UTR of *rpsJ* from the genomic DNA of Corynebacterium diphtheriae NCTC 13129, while appending SacI, PstI, KpnI, SalI, and NdeI sites to the DNA fragment (UTR-rpsJ). Second, the mCherry coding sequence of pCWU3 (43) was amplified from this vector using primers mCherry-F and mCherry-R (Table S3), which append NdeI, HindIII, BglII, XhoI, and BamHI to the amplified fragment. Both UTR-rpsJ and mCherry fragments were subcloned into pHS30 at SacI and HindIII sites, producing pCWU6 that has two multiple cloning sites (MCSs) (Fig. S6). Thus, insertion of DNA fragments into to the MCSs of pCWU6 results in the loss of mCherry expression, hence providing a convenient way to select cloned plasmids.

### (v) pEnvC_FLAG_.

The DNA fragment compassing *ftsX* and *envC* was PCR amplified from the genomic DNA of F. nucleatum ATCC 23726 using primers ftsX-F2 and com-EnvC-R. The PCR product was digested by KpnI and XhoI and subcloned into pCK-galK (44) (Fig. S6E). The resulting plasmid was used as the template for a reverse PCR using primers RE-envC-F and RE-envC-R (Table S3). The generated PCR product was gel purified and phosphorylated for ligation into a circular plasmid. Using this plasmid DNA as the template, primers com-FtsX-F and com-EnvC-R (Table S3) were used to PCR amplify the DNA fragment containing the *envC* coding sequence, while appending a dual hexahistidine/3×Flag tag. The *envC* fragment was digested with KpnI and XhoI, mixed with the *catP* promoter (see above) precut with SacI and KpnI, and then ligated into pCWU6 precut with SacI and XhoI.

### (vi) pFtsX_FLAG_.

To construct this vector, primer sets PcatP-F/R and com-FtsX-F/R (Table S3) were used to amplify the *catP* promoter from plasmid pHS30 and the *ftsX* coding region from the genomic DNA of F. nucleatum ATCC 23726, appending SacI/KpnI or KpnI/XhoI sites for cloning purposes, respectively. The PCR products were cut by SacI/KpnI or KpnI/XhoI and cloned into the SacI and XhoI sites of pCWU6.

To generate deletion constructs of FtsX, i.e., deletion of ECD1, ECD2, and D/E-rich domains, pFtsX_FLAG_ was used as the template for PCR amplification using the appropriate primer sets (Table S3) for the desired deletion. The PCR products were gel purified, followed by phosphorylation with T4 kinase and ligation with ligase. The resulting plasmids were confirmed by DNA sequencing and subsequently electroporated into the Δ*ftsX* mutant.

### (vii) pMinC.

Primers rpsJ-F and rpsJ-R containing SacI and KpnI sites were used to amplify the untranslated region of *rpsJ* from F. nucleatum ATCC 23726 using genomic DNA as the template. The *minC* sequence was amplified by using primers minC-F and minC-R, harboring KpnI and XhoI sites. Both fragments were subcloned into pCWU6 at SacI and XhoI sites.

### Development of a facile gene deletion method in F. nucleatum*.*

To make an in-frame deletion mutant of *galK*, the gene deletion vector pCWU5-ΔgalK was electroporated into F. nucleatum ATCC 23726, and integration of the plasmid into the chromosome was selected on TSPC agar plates supplemented with 5 µg ml^−1^ thiamphenicol (Thia). Thia-resistant colonies were cultured overnight in TSPC broth without antibiotics. The following day, 50-µl aliquots of cultures diluted 1,000-fold were plated on TSPC agar plates containing 0.25% 2-deoxy-d-galactose (2-DG). After 3-day incubation in the anaerobic chamber at 37°C, 10 randomly chosen 2-DG-resistant colonies were restreaked on TSPC agar plates containing 2-DG prior to colony PCR amplification using primers galK-F and galK-R (Table S3) to detect the loss of *galK* in these colonies.

Using the *galK* deletion mutant, which is Thia sensitive (Fig. S6C), as a parental strain, in-frame deletion mutants of *ftsX* and *envC* were generated. To create deletion constructs of *ftsX* and *envC*, 1.0-kb segments upstream and downstream of each gene were amplified by PCR using appropriate primers (Table S3). The upstream fragments were digested with SacI and KpnI, while the downstream fragments were digested with KpnI and SalI. These fragments were ligated into pCWU8 precut with SacI and SalI. The generated plasmids were confirmed by restriction enzyme digestion.

The generated plasmids with upstream and downstream arms for homologous recombination were then introduced into the Δ*galK* mutant by electroporation. Plasmid insertion into the bacterial chromosome by homologous recombination was selected by growth in the presence of Thia at 37°C. A Thia-resistant colony was cultured overnight in TSPC broth without antibiotics. The following day, a 100-µl aliquot of 1,000-fold-diluted cultures was plated on TSPC agar plates containing 0.25% 2-DG. Typically, ten 2-DG-resistant colonies were retested for their sensitivity to 2-DG and Thia. Thia-sensitive colonies were screened by PCR amplification for the loss of *ftsX* or *envC*.

### Tn*5* transposon mutagenesis of F. nucleatum and screening for biofilm-defective mutants.

We modified the commercially available EZ-Tn5 transposon system (Epicentre) for our purpose by cloning the chloramphenicol/thiamphenicol resistance cassette of pHS30 into pMOD-2<MCS> (Epicentre) to generate pMOD-*catP*. We next produced a transposome stock by mixing 0.8 µg of linearized Tn*5* transposon DNA—obtained by digestion of pMOD-*catP* with PvuII—mixed with 4 U of EZ-Tn5 transposase (Epicentre), and incubating the reaction mixture for 4 h at room temperature. Two microliters of the transposome (~200 ng) generated was then mixed with 200 µl of the F. nucleatum ATCC 23276 competent cells and incubated on ice for 10 min before electroporation. Electroporated cells were spread on TSP agar plates containing thiamphenicol. A library of approximately 24,000 thiamphenicol-resistant Tn*5* mutants were generated from the parental F. nucleatum ATCC 23726 strain from a single Tn*5* transposome reaction that was carried out in bulk, based on reaction efficiency determined from small-scale quantitative electroporation experiments.

In a pilot screen for biofilm-associated factors, 1,000 Tn*5* mutants were individually inoculated into 96-well plates containing 200 µl of TSPC broth in an anaerobic chamber at 37°C. After 48-h growth, 10 biofilm-defective mutants were picked by their inability to form biofilms on the bottom of the wells compared to the parental strain. These mutants were subsequently confirmed by a crystal violet biofilm assay (see below).

### Single-primer one-step PCR (SOS-PCR) mapping of Tn*5* insertion sites.

To identify genes targeted by Tn*5* insertions, a rapid and convenient mapping procedure was established for F. nucleatum based on a published procedure (25), which involves a single PCR consisting of three rounds of amplification and one transposon-specific primer. Briefly, 2-µl portions of overnight cultures of fusobacterial strains were added to a 50-µl PCR mixture consisted of 1.0× *Taq* RED Mater Mix kit (Apex BioResearch Product) and 0.4 µM FnTn5-F1 primer (5′-GCAAAAACATCGTAGAAATACGGTG-3′). The PCR program was started with 5 min at 95°C, followed by 20 cycles, with 1 cycle consisting of 30 s at 95°C, 30 s at 50°C, and 3 min at 72°C; this was immediately followed by 30 cycles, with 1 cycle consisting of 30 s at 95°C, 30 s at 30°C, and 2 min at 72°C and then 30 cycles, with 1 cycle consisting of 30 s at 95°C, 30 s at 50°C, and 2 min at 72°C. The PCR program ended with 10 min at 72°C. The generated PCR products were gel purified, and 50 ng of purified DNA was used for DNA sequencing using 25 ng of FnTn5-F2 primer (5′-GGCTTAAAACAAGGATTTT-3′). The generated sequences were analyzed against the genomic sequence of F. nucleatum ATCC 23726 (https://biocyc.org/) to identify Tn*5* insertion sites.

### Biofilm assays.

For monospecies biofilms, overnight cultures of fusobacterial strains were used to inoculate fresh cultures (1:100 dilution) in 3 ml of TSPC in flat-bottom 12-well plates (Greiner Bio-One). After anaerobic growth at 37°C for 48 h, the biofilms were gently washed twice with water prior to air drying. The biofilms were stained with 0.4 ml of 1% crystal violet for 10 min at room temperature, gently washed twice with water, and air dried prior to imaging with an AlphaImager (InnoTech). The assays were performed in triplicate and repeated at least two times.

Multispecies biofilms were cultivated following a previously published procedure (32). Briefly, overnight cultures of S. oralis grown in BHI supplemented with 0.5% glucose, A. oris grown in HIB, and F. nucleatum grown in TSP medium supplemented with 0.01% cysteine were each washed with YPTG medium (0.17% yeast base, 20 mM Na_2_HPO_4_-NaH_2_PO_4_ [pH 7], 0.1% tryptone, and 0.4% glucose). Aliquots of S. oralis cells (~10^7^ CFU) were grown in 3 ml of YPTG medium for 2 h at 37°C in 10% saliva-coated glass-bottom microwell dishes (35-mm diameter, 14-mm microwell; MatTek Corporation, MA). Unattached cells were then aspirated, and 2 ml of YPTG medium containing A. oris and F. nucleatum wild-type strain, Δ*ftsX* or Δ*envC* mutant (10^8^ CFU each) was added to the wells with adherent S. oralis cells. After 1 h of incubation at 37°C to allow cell-cell interaction, the medium was replaced with 3 ml of fresh YPTG medium, and the cultures were maintained in an anaerobic chamber for 48 h at 37°C. The biofilms obtained were washed twice with phosphate-buffered saline (PBS) and fixed with 0.5 ml of 4% formaldehyde for 2 h at room temperature. The biofilms were then washed twice with PBS and incubated at 55°C for 2.5 h in 1 ml of hybridization buffer (900 mM NaCl, 20 mM Tris-HCl [pH 8], 0.01% SDS, 20% formamide, and 5 mM ethylene diamine tetraacetic acid) containing 5 µg of probes labeled with fluorescein isothiocyanate (FITC) (green), Cy5 (red), and Pacific blue (blue). The probes were 16S rRNA-oligonucleotide probes specific for A. oris (GCTACCGTCAACCCACCC), F. nucleatum (CCCTAACTGTGAGGCAAG), and S. oralis (CCACAGCCTTTAACTTCAGA) (45). After the biofilms were washed with 1 ml of wash buffer (278 mM NaCl, 20 mM Tris-HCl [pH 7.5], and 0.01% SDS) and rinsed with 1 ml of 0.9% NaCl, they were analyzed by confocal laser scanning microscopy at a magnification of ×20. The experiments were performed in triplicate and repeated three times.

### Triton X-100 sensitivity assay.

Fifty microliters of 20% Triton X-100 was added to wells with a diameter of 0.3 mm created by punching TSPC agar plates with a sterile pipette tip. After detergent diffusion was complete, 10 ml of a 1.2% TSPC agar gel mixed with a 100-µl aliquot from a stationary-phase bacterial culture was overlaid onto the TSPC agar plates. The zones of inhibition were recorded after 72 h of incubation in an anaerobic chamber at 37°C.

### Western blotting analysis.

For immunoblotting, polyclonal antibodies against FtsX and HslJ, a predicted inner membrane protein (HMPREF0397_1592) used as a control, were generated from recombinant proteins. To clone both proteins, primers EXL1^FtsX^-F/R and H6-HslJ-F/R were used to PCR amplify the DNA region encoding the extracellular loop 1 of FtsX and the coding region of HslJ without the signal peptide sequence, respectively. The generated amplicons were treated with SspI and T4 DNA polymerase in the presence of only dGTP and then ligated into the expression vector pMCSG7 (46). The recombinant plasmids were introduced into E. coli BL21(DE3). Purification of the recombinant H6-FtsX and H6-HslJ were carried out by affinity chromatography based on a published protocol (47). The purified proteins were used for antibody production (Cocalico Biologicals, Inc.).

To detect FtsX in fusobacteria, log-phase cultures (optical density at 600 nm [OD_600_] of 0.6) of the wild-type strain and its derivatives were harvested by centrifugation. Cell pellets were washed twice with water and suspended in sample buffer containing sodium dodecyl sulfate (SDS). Samples were boiled 10 min and subjected to SDS-PAGE using Tris-glycine gradient gels (4 to 20%), followed by immunoblotting with the generated antibodies against FtsX and HslJ (anti-FtsX antibody diluted 1:1,000; anti-HslJ antibody diluted 1:5,000).

### Electron microscopy.

Transmission electron microscopy was performed according to a previously published protocol (48). Briefly, fusobacteria grown in TSPC broth were harvested by centrifugation and suspended in 0.1 M NaCl. A drop of bacterial suspension in phosphate-buffered saline was placed onto carbon-coated nickel grids and stained with 1% uranyl acetate. Samples were washed with water prior to imaging with a JEOL JEM1400 electron microscope.

For scanning electron microscopy, fusobacteria harvested at exponential phase were placed on coverslips and fixed with 2.5% (vol/vol) glutaraldehyde in PBS for 30 min at room temperature. After the cells were washed three times with PBS, they were dehydrated in a graded series of alcohol (30, 50, 70, 90, and 100% [vol/vol]) for 10 min for each alcohol solution. After optimal drying, samples were coated with a 5-nm metal film (Pt-Pd [80:20; Ted Pella Inc.]) using a sputter coater (Cressington sputter coater 208HR; Ted Pella Inc.) and imaged using a Nova NanoSEM 230 microscope (FEI). The scanning work distance was 5 mm, and the accelerating high voltage was 5 kV.

For cryo-electron microscopy, fusobacterial samples mixed with 10-nm gold particles, which were used as reference markers, were deposited onto freshly glow-discharged, holey carbon grids for 1 min. The grids were blotted with filter paper and rapidly frozen in liquid ethane, using a gravity-driven plunger apparatus as previously described (49). The frozen-hydrated specimens were imaged at −170°C using a Polara G2 electron microscope (FEI) equipped with a field emission gun and a direct detection device (Gatan K2 Summit). The microscope was operated at 300 kV with a magnification of 9,400, resulting in an effective pixel size of 5.2 Å at the specimen level. The tilt series images were aligned and reconstructed using an IMOD software package.

### Identification of FtsX- and EnvC-interacting proteins.

Cultures of the wild-type (WT) strain, Δ*ftsX* mutant expressing H6-Flag-tagged FtsX (X-FLAG), and Δ*envC* mutant expressing X-FLAG or H6-Flag-tagged EnvC (C-FLAG) were grown exponentially to an OD_600_ of ~0.6. Formaldehyde was added to 500 ml of cultures to the final concentration of 1.2%, and the treated or mock-treated cultures were maintained at 37°C for 20 min. The cross-linking reactions were quenched by the addition of 20 g glycine for 20 min at room temperature. Cell pellets were collected by centrifugation (8,000 × *g* for 10 min at 4°C), washed with 40 ml of PBS, and stored at −80°C until use.

The frozen pellets were subjected to membrane isolation based on published procedures (39, 50) with some modifications. Briefly, cell pellets were suspended in 8-ml portions of buffer A (50 mM Tris-HCl [pH 7.5], 150 mM NaCl, 1 mM EDTA) supplemented with 10 mM MgCl_2_, 10 U/ml DNase and RNase, and 0.1 ml of 1 M phenylmethylsulfonyl fluoride (PMSF). Cells were lysed by a combination of sonication and treatment with a French press. Membrane fractions were collected by centrifugation at 75,600 × *g* for 2 h at 4°C, and the pellets were suspended in 4 ml of buffer B (buffer A plus 2% Triton X-100 and 1.6% *N*-lauroylsarcosine) and incubated overnight at 4°C with rotary shaking. Finally, soluble membrane lysates were obtained by centrifugation at 26,700 × *g* for 2 h at 4°C.

FtsX and EnvC with dual His and FLAG tags in the membrane lysates were subjected to tandem affinity chromatography. First, the tagged proteins were purified by chromatography using nickel-nitrilotriacetic acid (Ni-NTA; Qiagen) based on a published protocol (51). Eluates from Ni-NTA purification were then mixed with 300 microliters of anti-FLAG M2 affinity gel (Sigma) and maintained in a rotary shaker for 6 h before loading onto a 2-ml gravity flow column. Bound proteins were washed 10 times with 2 ml of buffer A before being eluted from the column with 200 µl of elution buffer (buffer A containing 200 ng 3×FLAG peptide/µl). Eluted samples were concentrated to 100 microliters by a SpeedVac concentrator and mixed with an equal volume of 2× Laemmli sample buffer containing 5% (vol/vol) β-mercaptoethanol prior to SDS-PAGE with Tris-glycine gradient gels (4 to 20%), followed by immunoblotting with anti-FLAG. Gel pieces of interest were excised and subjected to protein identification by mass spectrometry (Proteomics Service Center, Institute of Molecular Medicine, The University of Texas Health Science Center at Houston [UTHealth]). Proteins identified from the WT and untreated samples were considered background.

For coimmunoprecipitation (co-IP) assays, membrane lysates from the Δ*envC* mutant expressing C-FLAG were obtained as mentioned above. Triton X-100 and *N*-lauroylsarcosine were removed with desalting columns (Bio-Rad), and the lysates were eluted in 4 ml of buffer C (150 mM NaCl, 50 mM Tris-HCl [pH 7.5], 1 mM PMSF). Equal volumes of membrane lysates (1.9 ml) were added to 100 µl of anti-FtsX or prebleed sera for 6 h at 4°C with rotary shaking, and then the membrane lysates plus antibody or sera were incubated with 300 µl of protein A agarose beads (Thermo Scientific) overnight. Protein A beads were collected by centrifugation at 600 × *g* and washed five times with buffer C. Bound proteins were eluted from the beads by boiling in 100 µl of 2× loading buffer without β-mercaptoethanol for 10 min. Eluted proteins were subjected to SDS-PAGE with 3 to 20% Tris-glycine gradient gels and immunoblotted with anti-FtsX or anti-FLAG.
