# Supplementary figures and images for: Forward Genetic Dissection of Biofilm Development by Fusobacterium nucleatum: Novel Functions of Cell Division Proteins FtsX and EnvC
Source: mBio. 2018 Apr 24;9(2):e00360-18. doi: 10.1128/mBio.00360-18 (PMC5915739; doi:10.1128/mBio.00360-18)

**A**

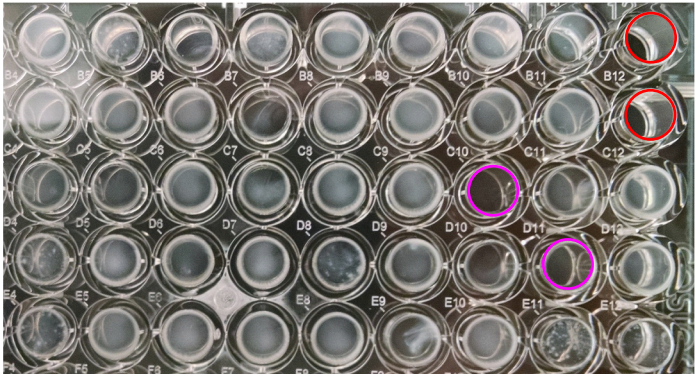

**B**

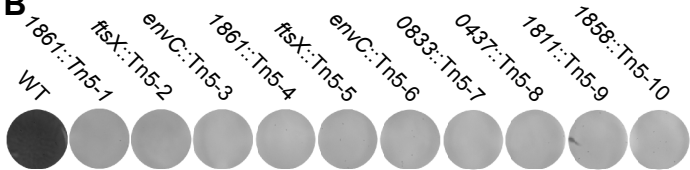

**C**

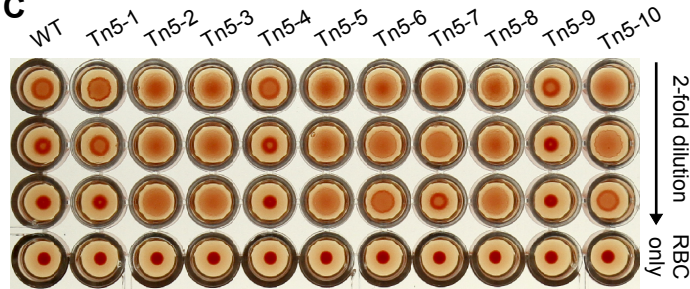

**D**

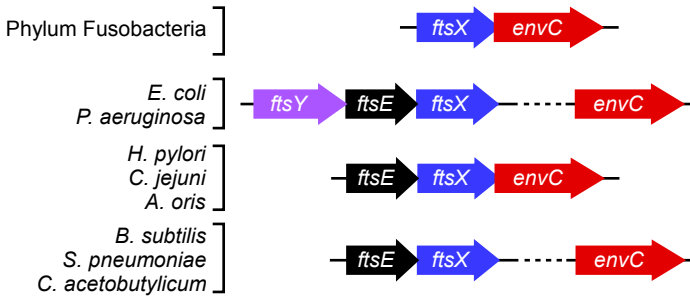

Figure S1: Wu et al.

Supplement: FIG S1 [file mbo002183846sf1.pdf]

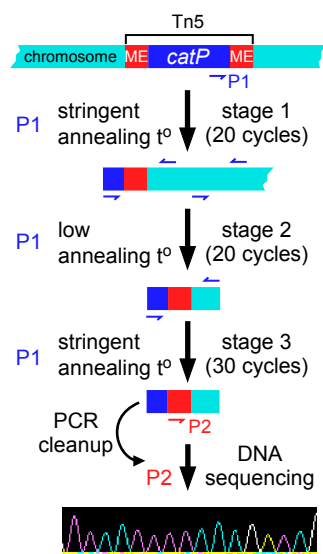

Figure S2: Wu et al.

Supplement: FIG S2 [file mbo002183846sf2.pdf]

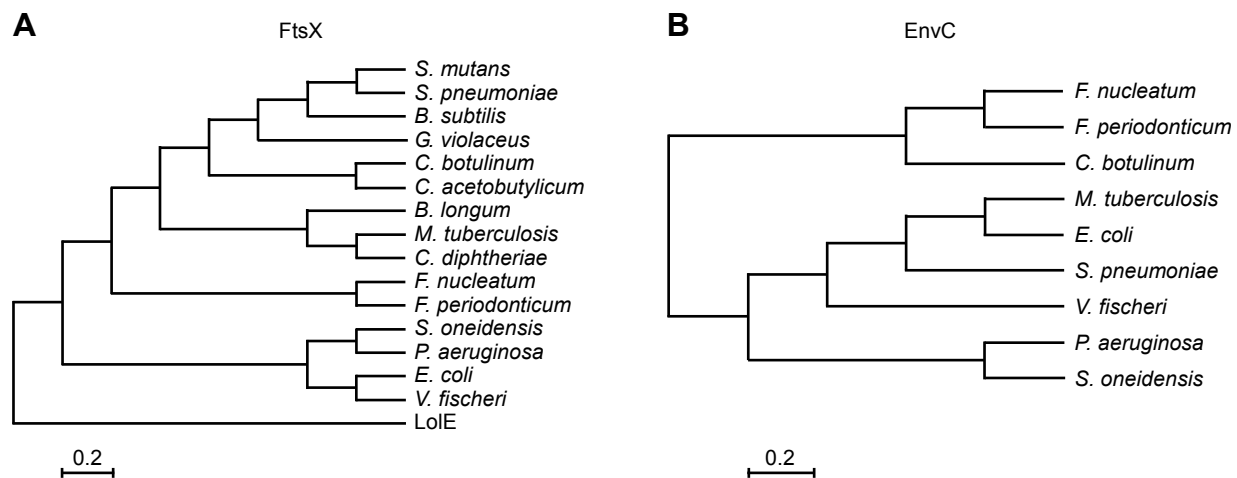

Figure S5: Wu et al.

Supplement: FIG S5 [file mbo002183846sf5.pdf]

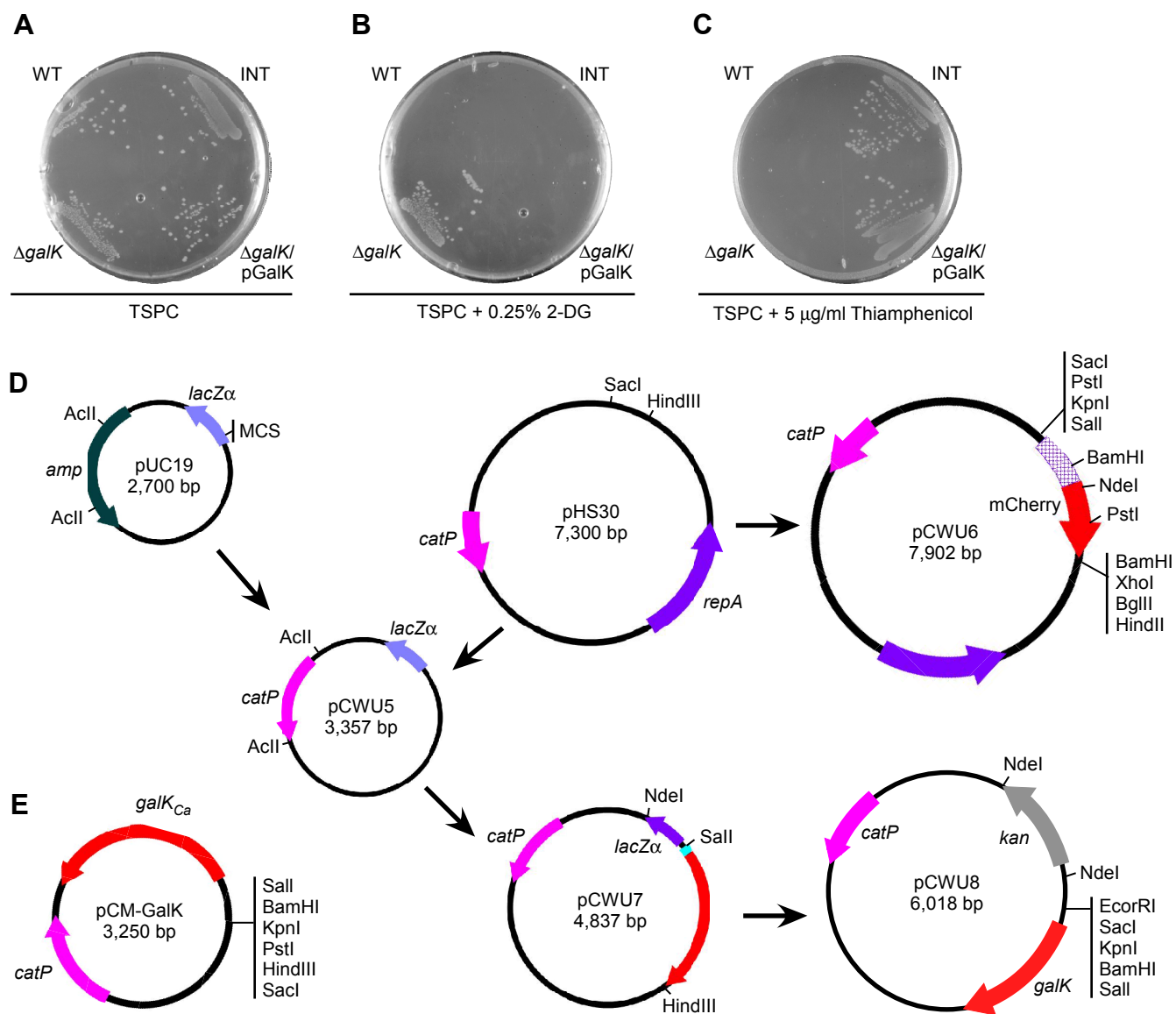

Figure S6: Wu et al.

Supplement: FIG S6 [file mbo002183846sf6.pdf]

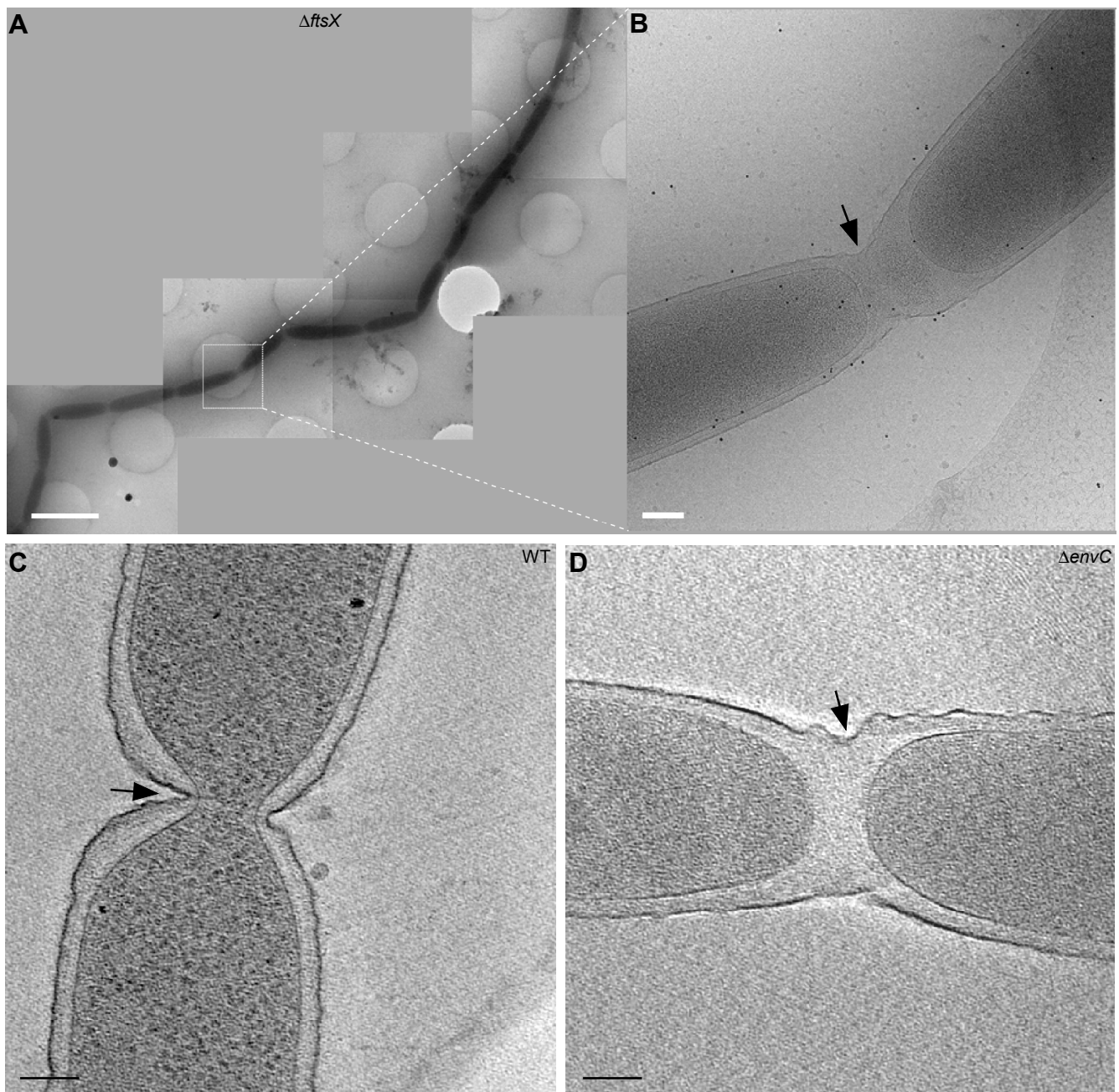

Figure S7: Wu et al.

Supplement: FIG S7 [file mbo002183846sf7.pdf]
