# Supplementary material for: Forward Genetic Dissection of Biofilm Development by Fusobacterium nucleatum: Novel Functions of Cell Division Proteins FtsX and EnvC
Source: mBio. 2018 Apr 24;9(2):e00360-18. doi: 10.1128/mBio.00360-18 (PMC5915739; doi:10.1128/mBio.00360-18)
Supplement: FIG S3 [file mbo002183846sf3.pdf]

```
F. nucleatum        1 MYK---------------------------------------------------------
F. periodonticum    1 MYK---------------------------------------------------------
E. coli             1 MNKRDAINHIRQFGGRLDRFRKSVGGSGDGGRNAPKRA-K----SSPK-PVNRKTNVFNE
P. aeruginosa       1 MSANDLPRG--------------P-EE-----GAPERKTREKPSQEQTDWSG-SFSAYLE
B. subtilis         1 MIK---------------------------------------------------------
M. tuberculosis     1 MRF---------------------------------------------------------
S. mutans           1 MIR---------------------------------------------------------
S. pneumoniae       1 MSLPLKMAV--------------SFV-----------------TNQKE-----SMDTMIS
C. diphtheriae      1 MKL---------------------------------------------------------
B. longum           1 MRA---------------------------------------------------------
S. oneidensis       1 MSDNAKLTR--------------SKLP----------------------ISG-RIVMFFI
C. acetobutylicum   1 MRV--------------------------------------------------------S
C. botulinum        1 MKI--------------------------------------------------------S
G. violaceus        1 MIQRVTT-----------------------------------------------------
V. fischeri         1 MAKRKPKNT--------------QGA--------------------------SNDGFWA

F. nucleatum        4 LFGYGLK-GIPYINRLKRRVFY---AVVITVVALNIFISFSLNLKSLTNEKIFNSFIVAD
F. periodonticum    4 LFGYGLK-DIPYINRLKNRVFY---IIVITIVSLNIFISFSLNLKKVSKETLINSFIIVD
E. coli            55 QVRYAFHGALQDLKSKPFATFLTVMVIAISLTLPSVCYMVYKNVNQAATQYYPSPQITVY
P. aeruginosa      40 SHRASLVDSLRRLFGHPFGSFFTCLVMGITLSLPMGLSLLLNNVERLGGSWQRAAQISLF
B. subtilis         4 ILGRHLRESFKSLGRNTWMTFASISAVTVTLILVGVFLVIMLNLNNMATNAEKQVEIKVL
M. tuberculosis     4 --GFLLNEVLTGFRRNVTMTIAMILTTAISVGLFGGGMLVVRLADSSRAIYLDRVESQVF
S. mutans           4 RFFRHLWQSIKNLKRNGWMTVAAVSSVTITLILVGIFAAFLLNTEKLASGIEKNVQINTY
S. pneumoniae      25 RFFRHLFEALKSLKRNGWMTVAAVSSVMITLTLVAIFASVIFNTAKLATDIENNVRVVVY
C. diphtheriae      4 --GFVFREAFRGLGRNITMTIALIITTAISLALLATGFLVTNMTKETKDIYLDRVEVMVQ
B. longum           4 RF--ILSETWANLTRNLSMLLSLTLVTFISFLFIGASVLTQAQITKAKGDWYDKVEVVVW
S. oneidensis      24 RHVQQAMASMGELWRSPVSSLMTMAVLGVSLSLPAALQVLVKNAETITSSWNSAAEISLF
C. acetobutylicum   5 TLKLFFIDALKSLKRNKTISTAAATVAATLFILGVCLLVLLNVKTGISDVRSKVQVQVY
C. botulinum        5 TLKYFVVDSIKGLRRHKTLSTASIATVAATLFILGVFILSIMNVKQAVTEVESKVEATIV
G. violaceus        8 QVDYLLREAFTGLRRNLWMIWSAVSTLAVLLFLLGLGLRASWQLQDAVSDLGSRLEISIY
V. fischeri        20 THKKQAKLSFQELLRRPLGNMLTLAVIAMSLTLPSSMYLIGKNLTIVASKWQAPSQVSLY

F. nucleatum       60 LQNNLNQ-----------------D----KKNEIEKYILGINGVRSVRFMDKFESFKN-L
F. periodonticum   60 LQNNLDE-----------------E----KRNEIEKYILTIDGVRSVRFMDKSESFKN-L
E. coli           115 LQKTLD-----------------DD----AAAGVVAQLQAEQGVEKVNYLSREDALGE-F
P. aeruginosa     100 LDLKTS-----------------EN----QGQDLREQIERLPDVIEAQLISREQALSE-L
B. subtilis        64 IDLTADQKA--------------------QDKLQNDIKELKGIQSVTFSSKEKELDQ-L
M. tuberculosis    62 LTEDVSANDSS----------CDTT----ACKALREKIETRSDVKAVRFLNRQQAYDD-A
S. mutans          64 LQVDSKDNVKSYVDSNDANKTVNNP----DYHKVYDQIKKIEHVKSITFSSKDEQLEK-L
S. pneumoniae      85 IRKDVEDNSQTI-EK-E-GQTVTNN----DYHKVYDSLKNMSTVKSVTFSSKEEQYEK-L
C. diphtheriae     62 LNEDISANDKD----------CSSQ----ACRDVRDKLDGADGIETVTYRSRQQSYDR-F
B. longum          62 LCPDGTSQSANCASG-K---SPSANEITALQKTIRDELND--VVSNIDYVSKQDFYDNTF
S. oneidensis      84 IDENRS-----------------EQ----TIQSLLTRIRTYPEVEKVQYIDRNQALEE-F
C. acetobutylicum  65 FKDDITIDE--------------------QKIVLNKLADVPGITGIKFESKSDALAK-F
C. botulinum       65 LKDDIKTEQ--------------------EKAIKDKINSVAGVEKVTYESKKDALEK-F
G. violaceus       68 LDPGVRA----------------------AAVEPDLRTLVGVDEIKSVSKEQAWAA-L
V. fischeri        80 LQQDVA-----------------ET----KVQTLKSELERWKEIEAVQYISPQEGLKE-L

F. nucleatum       98 QNELNIS---I---PESSNPLTDSLVISVKDP----TLLGQIQETIE-----SREEVKEV
F. periodonticum   98 QNELNIS---I---PEASNPLTDSLIVSVKSA----ELMNGVQEIIE-----AREEVKEV
E. coli           153 RNWSGFGGA-L--DMLEENPLPAVAVVIPKLDFQGTESLNTLRDRIT-----QINGIDEV
P. aeruginosa     138 QEQSGLGEA-L--KELPENPLPPVISVTPKQI--DRAGLEALRQQLA-----ELPHVQQA
B. subtilis       102 VDSFGDSGKSLT-MKDQENPLNDAFVVKTTDP----HDTPNVAKKIE-----KMDHVYKV
M. tuberculosis   107 IRKFPQFK-D-V-A--GKDSFPASFIVKLENP----EQHKDFDTAMK-----GQPGVLDV
S. mutans         119 KKTMGDDW-NLF--DGDSNPLYDVYIVQTTSP----SEVKTVAKAIG-----KISGVDSV
S. pneumoniae     137 TEIMGDNW-KIF--EGDANPLYDAYIVEANAP----NDVKTIAEDAK-----KIEGVSEV
C. diphtheriae    107 VEVFKDTDPQLV-AETSPDALPAALHVRLEDP----LDTKPLD-QVR-----DMEQVDTI
B. longum         116 TKQYPNGE-FQG-RTLTADDMQDSLWLKLKDP----TKYQVVSEVLS-----GKEGVEDV
S. oneidensis     122 QRLSGFGEA-L--AYLDKNPLPAVITVTPTQRYSTPVGARELL---T-----KLEREPEI
C. acetobutylicum 103 KQQLGNDNKTLVEGMDTRNPMPNSYVVSVSDS----DYASGVVKALKDKNGSTLDGIEKI
C. botulinum      103 KKQLGENNKSLAEGLEKENPLPNSIIVRVEKP----ELVSKVVGSIK-----DMEGIDQI
G. violaceus      103 KKDLGVRS-DPG-ESLGGNPLVDSLRVRIAQP----EAVAPLAAQIQ-----QIEGVEEV
V. fischeri       118 SEHSGFEQA-L--TLLDSNPLPAVLIVSPKTEWQGTEQVNQLVNRLK-----QQSYVNEV
```

```
F. nucleatum      143 YKD---ESYLKQSKEQGFITSIAQIGSGVFSFFIALITIIIFNFGVAIE--FLNNANTGL
F. periodonticum  143 YKD---EPYLKQSQEQSDIIRIAQIGSAVFSILIALVTIVIFNLGVAIE--FLNNANTGL
E. coli           205 RMD---DSWFARLAALTGLVGR--VSAMIGVLM---VAAVFLVIGNSVRLSIFAR-RDSI
P. aeruginosa     188 QLD---LVWVERLSAILKLGER--FVFGLTILL---VLTLLLVVGNTIRLHIENR-RNEI
B. subtilis       152 TYG---KEEVSRLFKVVGVSRN--IGIALIIGL---VFTAMFLISNTIKITIFAR-RKEI
M. tuberculosis   153 LNQ---KELIDRLFAVLDGLSN--AAFAVALVQ---AIGAILLIANMVQVAAYTR-RTEI
S. mutans         167 AYG---GTDTDRIFGLANFVRT--WGLAGTGLL---VLVAIFLISNTIRITILSR-RNEI
S. pneumoniae     185 QDG---GANTERLFKLASFIRV--WGLGIAALL---IFIAAFLISNTIRITIISR-SREI
C. diphtheriae    156 VDQ---VDDLRGATDNLDAIRN--STFIFAAIQ---AIAAIFLIVNMVQIAAFNR-REEI
B. longum         165 TDQ---RQIFDPVFAVLNRATA--VTAVLAGVM---VVVAILLTGTTIRMSAASR-RTET
S. oneidensis     171 SFGRLDIEWLERLQAVVRLLER--TVMAIAALL---VLAVVLVIGNTIRLAIMNR-RSEI
C. acetobutylicum 159 QDG---RELVNKITTITNTVQW--VGIAIFIIL---AGVSLFLIGNTIKLTVYSR-RREI
C. botulinum      154 KDG---KEIVDKITKITNTLKW--MGVVLFLIL---IGVSLFLIGNTIKITVYSR-KREI
G. violaceus      152 SYG---SEAAQRLDQIQQAMRW--VGLALTAVL---GVATVAVITSTIRLIVQSR-RKEI
V. fischeri       170 RLD---DDWLARLDAIKHVAVV--VATTLAILM---FVAVFLIVGNTLRFNVLEQ-KDEI

F. nucleatum      198 DYAENIRKSKIRNLLSFTMSTVI--------GTLIFFNIYVLFRKHV-SHAN--FNSSML
F. periodonticum  198 DYRENIRSSKLKNLIPFSMASVV--------ATLIFFNIYIFFRKYV-INAN--FDSSLL
E. coli           256 NVQKLIGATDGFILRPFLYGGALLGFSGALLSLILSEILVL-RLSSA-VAEVAQVFGTKF
P. aeruginosa     239 EVIKLVGGTDGYVRRPFLYMGALYGLGAGILSWALLAYSLN-WLNGS-VVNLSGLYGSDF
B. subtilis       203 EIMKLVGATNWFIRWPFFLEGLLLGVFGSVIPIALVLSTYQYVIGW-VVPKVQGSFVSLL
M. tuberculosis   204 GIMRLVGASRWYTQLPFLVEAMLAATMGVGIAVAGLMVVRALFLENA-LNQ---FYQANL
S. mutans         218 QIMRLVGAKNGYIRTPFFFEGAWVGLIGAIIPAVVVGYLYIFAFEQ-FNPNLAAQNLSLY
S. pneumoniae     236 QIMRLVGAKNSYIRGPFLLEGAFIGLLGAIAPSVLVFIVYQIVYQS-VNKSLVGQNLSMI
C. diphtheriae    207 SIMRMVGASRWYTQAPFVIEAMVAALFGAILSGLALFGGKMWVVDKT-LKG---LYDSQL
B. longum         216 EIMRYVGASNWTIRLPFILEGAIASLIGSVLSCLMLSAIVNVFVTGWLAKSVT--WIP-Y
S. oneidensis     225 EVMKLVGATEAFIQRPFLYTGIWYGVIGGILAWVIINLLVW-YLDSA-LAELLGLYGSQL
C. acetobutylicum 210 GIMKYIGATDWFIRLPFVIEGMIIGIAGALVTILVVYNLYRVLFNK-IRSSFL--TMNII
C. botulinum      205 GIMKYIGATDWFIRWPFVFEGIIIGILGAIIAIVLLYYGYKAAYAK-ASVGLI--FVSLL
G. violaceus      203 EVMQLVGATPLRISMPFILEGLAFGIAGALIAWGLIEATSRVVAQKQ-LEL---LPFLQW
V. fischeri       221 QVMKLVGATNTFILRPYLYTGMWFGLLGGFVAWLLTAIITV-TLNGA-VDNVAVLYDSIF

F. nucleatum      247 S---LKEIILWHFGAIVILNLLVWLIPANVGRIEYAEEEDEDYDEFDDEFYEE----DGD
F. periodonticum  247 S---LKEIFLWHIGAIGILNFLVWIIPANLGRIEYEEENDDDLE---YEFYEDEDKKDEF
E. coli           314 D---INGLSFDECLLLLLVCSMIGWVAAWLATVQHLRH-------FTPE-----------
P. aeruginosa     297 G---LQGVPLDDGLSLTVGAVLLGWVGAWLAVARHLRE-------LAPR-----------
B. subtilis       262 P---YNPFVFQVSLVLIAIGAVIGVWGSLTSIRKFLR-----------V-----------
M. tuberculosis   260 IAKVDYADILFITPWLLLLGVAMSGLTAYLTLRLYVR-----------R-----------
S. mutans         277 E---PSPFIFYLIGAMFIVGILIGALGSILSMRRFLK-----------I-----------
S. pneumoniae     295 S---PDLFSPLMIALLFVIGVFIGSLGSGISMRRFLK-----------I-----------
C. diphtheriae    263 IARVSNADIWAVAPVVAVIGIIFAAITAQATLRWYVR-----------K-----------
B. longum         273 V---NQLTVLVISPFLVVGAILLSIIASTISLRRYLR-----------A-----------
S. oneidensis     283 E---MKSLTFTELLQLVGLASFLGWLGSYLSVRQHLRS-------IEPS-----------
C. acetobutylicum 267 Q---PSYVLTFMSWEFILAGMFIGALGSIVVIRKFLD-----------V-----------
C. botulinum      262 N---PSVVLSSVLWIFVLVGIVIGAIGSILSIRKFLS-----------V-----------
G. violaceus      259 --QPSEPAAFTLPLILLGVGVALGMLGSLIAVRRAI------------R-----------
V. fischeri       279 R---LVGLTWDESLLLLMLSSFLGLLAARISVLRHLKE-------IEPV-----------

F. nucleatum      300 YDEFEDDED--
F. periodonticum  301 YDEFEDEDENY
E. coli               -----------
P. aeruginosa         -----------
B. subtilis           -----------
M. tuberculosis       -----------
S. mutans             -----------
S. pneumoniae         -----------
C. diphtheriae        -----------
B. longum             -----------
S. oneidensis         -----------
C. acetobutylicum     -----------
C. botulinum          -----------
G. violaceus          -----------
V. fischeri           -----------
```

Figure S3: Wu et al.

Bacterial FtsX homologs. Shown is an alignment of amino acids of FtsX-like proteins. The GenBank accession codes are as follows: WP_005903510.1 (*Fusobacterium nucleatum*); WP_005974130.1 (*Fusobacterium periodonticum*); AAN82691.1 (*Escherichia coli*); WP_070142117.1 (*Pseudomonas aeruginosa*); WP_009968247.1 (*Bacillus subtilis*); CAA49620.1 (*Mycobacterium tuberculosis*); NP_721693.1 (*Streptococcus mutans*); CXG39040.1 (*Streptococcus pneumoniae*); WP_014318805.1 (*Corynebacterium diphtheriae*); WP_011068337.1 (*Bifidobacterium longum*); WP_011074182.1 (*Shewanella oneidensis*); WP_010963820.1 (*Clostridium acetobutylicum*); WP_011140650.1 (*Gloeobacter violaceus*); WP_005421374.1 (*Vibrio fischeri*). The multiple sequence alignment is generated using T-coffee (http://tcoffee.crg.cat/apps/tcoffee/do:regular).
