# Supplementary material for: Forward Genetic Dissection of Biofilm Development by Fusobacterium nucleatum: Novel Functions of Cell Division Proteins FtsX and EnvC
Source: mBio. 2018 Apr 24;9(2):e00360-18. doi: 10.1128/mBio.00360-18 (PMC5915739; doi:10.1128/mBio.00360-18)
Supplement: FIG S4 [file mbo002183846sf4.pdf]

```
F. nucleatum       1 MMNFMKKMEIMMSLKM-MKIKTILVFFLLSAS--IYPASKSVKD-MNKRLKN-------I
F. periodonticum   1 MKT-----------------KTILTFFLLSAS--IYPASNSVKD-MNKRLKN-------I
E. coli            1 MTRAVKPRR--------FAIRPIIYASVLSAGV-LLCAFSAHAD-ERDQLKS-------I
P. aeruginosa      1 MLRLLP--------------LLLS-LACLAP---AFA--DERAD-TQRQLEQ-------T
M. tuberculosis    1 MAHKHVFRT--HSRRVSPGVRTLLAALLLPVSFAVSAANTTTADASKNELKN-------L
S. pneumoniae      1 MLRFGVNQ----------KTSSLLLTALLSCGL-LIF--SPV-S-QSSDLNQ-------I
S. oneidensis      1 MSTRLPV-----------KASIIAGFMMLSFS--VYA--SNLEK-RQSELKS-------I
C. botulinum       1 MKKSLIKI----------VTIISLSCFIFESPI-VYA--TNSSD-IKDQINNNNQAIDNL
V. fischeri        1 MNISLLNNS--------FSCRRLATTALVCLA--FSSPILAH-A-SDAELKG-------M

F. nucleatum      50 DKEIEKKNTRIKAIDTETSKLEKMIKELEEEIKKLEHERKEIEDEITVVKKNIDYSRKNL
F. periodonticum  34 DKEIEKKNTRIKAIDTETSKLEKMIKELEEEIKKLEHEREEIEDEITVVKKNIDYSRKNL
E. coli           44 QADIAAKERAVRQKQQQRASLLAQLKKQEEAISEATRKLRETQNTLNQLNKQIDEMNASI
P. aeruginosa     33 QKDIGELKKLLDGIQQEKSGVQKQLKSTETEMGDLEKQIKALQDELDKSEAELKRLDGEK
M. tuberculosis   52 QLNIAEKEKSVKEQQGKRANLLTQLKNQEKDIAAAGRELYSTQKTLKQLDSEITTLSAEI
S. pneumoniae     39 QKQIKQQESKIEKQKREQAKLQANLKKHESKINSVEGELLETEISLKEIRKQIADADKQL
S. oneidensis     38 QAQINQQQSALKNTSKQREKLLALLRSDEEAIAAAAKKVNSTKTSLAQIDNTLAELKQRQ
C. botulinum      47 QNEKDKIQNQVNNVNKELDKITEQMEAKNKELEKSSKKVNEFQSKIDSLQAEIDSVQAKI
V. fischeri       42 KQEISRQSSVLNKQKKELSSLQNSLKKHEVSIANASKKIRNAEQELTTLKSSISSLKQQQ

F. nucleatum     110 EISEVEHNRK--------------ESE-FVAKIIAWDKYSKIHRKEIDEKVLLTK-NYRE
F. periodonticum  94 EISEVEHGRK--------------ESE-FVAKIIAWDKYSKIHGKDIDEKVLLTK-NYRE
E. coli          104 --AKLEQQKA--------------AQERSLAAQLD-AAFRQ--GEHTGIQLILSG-EESQ
P. aeruginosa     93 --KKLQDARI--------------EQQRLLAIQAR-AAYQS--GREEYLKLLLNQ-EHPE
M. tuberculosis  112 --KQLDAKQK--------------AQRALLSKQLD-AAFRQ--GRHQGIELIFKG-EEGQ
S. pneumoniae     99 --KQLEKQER--------------EQKARLAKQID-IIYRS--GINPSLIERMFA-QDPT
S. oneidensis     98 --EELESLKV--------------SQQNTLSKQLS-SAYLA--GNHDYTKMMLNQ-QSPA
C. botulinum     107 --NEAEEEIVKKEELIVQKENEAEERENMLGLRIR-NYYKN--DMTSQMLAFLVSSDGLS
V. fischeri      102 --SELTQQQI--------------GQTEILKDLLV-NYYLT--SRNNQLSNVLSG-DDVT

F. nucleatum     154 MLHGDLQRMGHI----EKVTGSIKEVKEKIEAEKRKLDRLEAELRENLRKSDIKKEEQKK
F. periodonticum 138 MLHGDLQRMGHI----EKVTGSIKEVKEKIEAEKRKLDRLEAELRENLRKSDAKKEEQKK
E. coli          144 RGQRLQAYFGYLNQARQETIAQLKQTREEVAMQRAELEEKQSEQQTLLY-------EQRA
P. aeruginosa    133 KFSRTLTYYDYINKARLEQLASFNETLRQLANVEQDISAQKAEQLSKQG-------ELDS
M. tuberculosis  152 RDERILAYYTYIGAAREKTIAELEETTQQLHARRQTEQQKRAEHKSLLG-------KQQT
S. pneumoniae    139 KAERMKVYYQHLNQVRIEMINNLKATQAQIAVQKKAVLSQQKNHRNQLS-------TQKK
S. oneidensis    138 TIERMLAYYQYLNKARMKSINELKQTITELDEIKVTQTSKQKQLTTLMA-------EQQV
C. botulinum     162 SFIGNIYNMKKILDTDKKLIDEVNSIKEALNNEKKLLEEKIVELDEEKV-------EIKN
V. fischeri      142 KMDRMTQYAQRISEARVGAISQLEFTNMQLEEKEADLLKKQQRQNELLA-------QYKK

F. nucleatum     210 LKEKL-------QVEKKGHQSSIEKLKKEKQRIS------KEIERIIR-----ENARRAA
F. periodonticum 194 LKEQL-------QVEKKGHQSSIEKLKKEKQRIS------REIERIIR-----ENARKAA
E. coli          197 QQAKL-------TQALNERKKTLAGLESSIQQGQQQLSELRANESRLR-----NSIARA-
P. aeruginosa    186 RREAL-------AATRKERQQALAKLNSDYRERDQKLKSRQQDQAREAAAAAERERQRA-
M. tuberculosis  205 EKKKL-------DRAQTARKSTLTELERSLKADQKDLLVMRENESQLR-----NKIAKA-
S. pneumoniae    192 QQQAL-------QKAQQEHQSTLNELNKNLALDQDKLNALKANEQALR-----QEIQRA-
S. oneidensis    191 QSKRL-------NQEQDQRQLTLNELQRTLNTKGAELEQLQIEEASLK-----RVVEQA-
C. botulinum     215 KQQELVNAQKEFVDEQNKYLAQMNDLKGIESKKQSMINSLSDKERELQ-----EKIGDL-
V. fischeri      195 EKVTL-------QTSQNKRKKTVSSIRKRISNENSYLSELQQNEKRLK-----TAIAKA-
```

```
F. nucleatum    252 EKAA--R-EKAAKEAAKNKGKG-S-KRSGGTKVTTTT-VDMPKISNPE-----AY-KRIG
F. periodonticum236 EKAA--R-EKAAREAAKNKGKS-AGKGSGETKVTTTT-VDMPKISNPE-----AY-KRIG
E. coli         244 EAAAKARAEREAREAQAVRDRQKEATRKGTT-YKPTE-SEKSLMSRTG-----GLGAPRG
P. aeruginosa   238 LAAE------------RERARQQQAAPGRVT-SPPREPAPGPLVSSTGAVYGGAFGSARG
M. tuberculosis 252 EREAKARAEREAREAARIREKQAQAKKKGST-YTPTQ-DERSLMSRTG-----GLGRPAG
S. pneumoniae   239 EQAAHEQE-KREREALAQRQKA-EEKRTSKP-YQPTV-QERQLLNSTS-----GLGAAKK
S. oneidensis   238 LKAM------------RD-------------------------N-----PSMEGFDKQGG
C. botulinum    269 TDFN---------Q--DLQNK---------------------LDDLFNGLDGGNSGNSQG
V. fischeri     242 KAKN--N-------------------------------------VPMD-----GIGRQKG

F. nucleatum    300 K-TIKPLNG-QIVVYFGQKK---AG--VV-ESNGIEIKGKLGNPVVASKAGTVIYADKFQ
F. periodonticum285 K-TIKPLNG-QIVVYFGQKK---AG--VV-ESNGIEIKGKLGNPVVASKAGTVIYADAFQ
E. coli         297 Q-AFWPVRG-PTLHRYGEQL---QG--EL-RWKGMVIGASEGTEVKAIADGRVILADWLQ
P. aeruginosa   285 K-LPWPVNG-RVVARFGSQR---GDDPRA-KWDGVLISASAGSTVRAVHGGRVVFADWLR
M. tuberculosis 305 Q-AIWPVRG-PLLHRFGDSI---SG--EL-RWKGMVIGAPEGTQVKAVADGRVLLADWLQ
S. pneumoniae   290 Q-YSLPVSG-SILHTFGSIQ---AG--EV-RWKGMVIGASAGTPVKAIAAGRVILAGYLN
S. oneidensis   256 K-LKWPTKG-RVSASFGSPR---SG--QV-VWKGTMLSAPEGQNIRAVSGGKVIYADWLK
C. botulinum    297 ETFLRPTSG-RVTSEYGPRTHPISG--QSGFHTGIDLASPSGTPIKASKSGTVVYSGWQG
V. fischeri     258 R-LPWPISNPKTLHSFGTKQ---TG--QL-TWKGMVLAGDYGTPVKAVYSGKVVFADWLR

F. nucleatum    352 GLGKVVMIDYGGGIIGVYGNLLAIKVNLNSKVSSGQTIGVLGLSSD-KEPNLYYELRANL
F. periodonticum337 GLGKVVMIDYGGGIIGVYGNLLAIKVNINSKVSSGQTIGVLGLSSD-KEPNLYYELRANL
E. coli         349 GYGLVVVVEHGKGDMSLYGYNQSALVSVGSQVRAGQPIALVGSSGGQGRPSLYFEIRRQG
P. aeruginosa   339 GAGLLVILDHGGGYLSLYGHNQSLLKDAGDTVKAGDPIATVGTSGGQSSPAVYFAIRHQG
M. tuberculosis 357 GYGLMVVVEHGKGDMSLYGYNQSALVNVGDQVKAGQPIALVGSSGGQERPGLYFEIRRQG
S. pneumoniae   342 GYGYMVIVKHGETDLSLYGFNQTVSVKVGQLVSAGQVIAQVGNTGEISRSALYFGISRKG
S. oneidensis   308 GFGMVMVIDHGKGYMSLYGHAQTLLKSPGEMVKTGEAIALVGRSGGQTEPGLYFEIRYKG
C. botulinum    354 GYGQVVIIDHGGGYRTLYAHCSKLNVVKNQKVSRGQVVALVGSTGNSTGPHLHFEVRVNN
V. fischeri     311 GYGLMVLIDHGKGDMTLYGYNQSLMKKEGDKVRAGETIAVVGDSGGQDRPSLYFEIRRNS

F. nucleatum    411 RPIDPIPTF----
F. periodonticum396 RPIDPIPTF----
E. coli         409 QAVNPQPWLG--R
P. aeruginosa   399 RPADPTTWCRAQG
M. tuberculosis 417 RTVNPQPWLG--R
S. pneumoniae   402 TPVNPAGWV---R
S. oneidensis   368 QAVDPAKYCR---
C. botulinum    414 KHQNPRKYVPI--
V. fischeri     371 KAQNPRSWLR--R
```

Figure S4: Wu et al.

Bacterial EnvC homologs. Shown is an alignment of amino acids of EnvC-like proteins. The GenBank accession codes are as follows: EFG94960.1 (*Fusobacterium nucleatum*); WP_099960967.1 (*Fusobacterium periodonticum*); WP_061354207.1 (*Escherichia coli*); WP_043091244.1 (*Pseudomonas aeruginosa*); SGD49707.1 (*Mycobacterium tuberculosis*); CVP50706.1 (*Streptococcus pneumoniae*); WP_011070463.1 (*Shewanella oneidensis*); WP_049177113.1 (*Clostridium botulinum*); WP_026029412.1 (*Vibrio fischeri*). The multiple sequence alignment is generated using T-coffee (http://tcoffee.crg.cat/apps/tcoffee/do:regular).
