# Supplementary material for: Forward Genetic Dissection of Biofilm Development by Fusobacterium nucleatum: Novel Functions of Cell Division Proteins FtsX and EnvC
Source: mBio. 2018 Apr 24;9(2):e00360-18. doi: 10.1128/mBio.00360-18 (PMC5915739; doi:10.1128/mBio.00360-18)
Supplement: TABLE S1 [file mbo002183846st1.pdf]

Table S1A: FtsX-associated proteins identified by mass spectrometry

N[(a)]	Protein ID	Predicted Function
1	HMPREF0397_1429	FtsX
2	HMPREF0397_1026	Tetratricopeptide repeat (TPR) family protein
3	HMPREF0397_1428	EnvC
4	HMPREF0397_1393	MORN repeat variant protein
5	HMPREF0397_1238	Hypothetical protein
6	HMPREF0397_0968	TPR protein
7	HMPREF0397_1681	Cation ABC superfamily ATP binding transporter
8	HMPREF0397_1477	Hypothetical protein
9	HMPREF0397_1313	MORN repeat protein
10	HMPREF0397_0730	Hypothetical protein
11	HMPREF0397_0327	Ser/Thr protein kinase
12	HMPREF0397_1293	AMP-binding enzyme
13	HMPREF0397_1662	SPFH domain/Band 7 family protein
14	HMPREF0397_0789	Hypothetical protein
15	HMPREF0397_0605	Hypothetical protein
16	HMPREF0397_0711	Hypothetical protein
17	HMPREF0397_1450	Hypothetical protein
18	HMPREF0397_1175	Hypothetical protein
19	HMPREF0397_0745	Hypothetical protein
20	HMPREF0397_0565	Peptidase, M23 family; NlpD
21	HMPREF0397_2044	Hypothetical protein
22	HMPREF0397_0259	Hypothetical protein
23	HMPREF0397_1251	Hypothetical protein
24	HMPREF0397_1028	Transpoter MotA
25	HMPREF0397_0255	Hypothetical protein
26	HMPREF0397_0561	Hypothetical protein

[(a)]The order is based on the abundance level of proteins identified by LC-MS/MS. Only proteins with 4 or more tryptic peptides detected by mass spectrometry are listed.

Table S1B: EnvC-associated proteins identified by mass spectrometry

N[(a)]	Protein ID	Predicted Function
1	HMPREF0397_1428	EnvC
2	HMPREF0397_1026	TPR protein
3	HMPREF0397_1393	MORN repeat variant protein
4	HMPREF0397_1313	MORN repeat protein
5	HMPREF0397_0075	DdpA
6	HMPREF0397_0704	Hypothetical protein
7	HMPREF0397_0968	TPR protein
8	HMPREF0397_1429	FtsX
9	HMPREF0397_0675	N-acetylmuramoyl-L-alanine amidase, AmidD3
10	HMPREF0397_1477	Hypothetical protein
11	HMPREF0397_0605	Hypothetical protein
12	HMPREF0397_0789	Hypothetical protein
13	HMPREF0397_1251	Hypothetical protein
14	HMPREF0397_0259	Hypothetical protein
15	HMPREF0397_1686	ABC transporter, substrate-binding protein
16	HMPREF0397_2085	MORN repeat protein
17	HMPREF0397_0202	von Willebrand factor type A domain protein
18	HMPREF0397_0711	Hypothetical protein
19	HMPREF0397_2044	Hypothetical protein
20	HMPREF0397_0561	Hypothetical protein
21	HMPREF0397_0107	TPR protein
22	HMPREF0397_0542	MORN repeat protein
23	HMPREF0397_1623	Hypothetical protein
24	HMPREF0397_0509	Cyclically-permuted mutarotase family protein
25	HMPREF0397_2045	Hypothetical protein
26	HMPREF0397_1662	SPFH domain/Band 7 family protein
27	HMPREF0397_0565	Peptidase, M23 family; NlpD

[(a)]The order is based on the abundance level of proteins identified by LC-MS/MS. Only proteins with 4 or more tryptic peptides detected by mass spectrometry are listed.
