# Supplementary material for: Forward Genetic Dissection of Biofilm Development by Fusobacterium nucleatum: Novel Functions of Cell Division Proteins FtsX and EnvC
Source: mBio. 2018 Apr 24;9(2):e00360-18. doi: 10.1128/mBio.00360-18 (PMC5915739; doi:10.1128/mBio.00360-18)
Supplement: TABLE S2 [file mbo002183846st2.pdf]

Table S2: Bacterial strains and plasmids used

Strain & Plasmid	Description	Reference
Strain		
F. nucleatum 23726	Parental strain	(1)
F. nucleatum CW1	Δ*galK*; an isogenic derivative of 23726	This study
F. nucleatum CW2	Δ*ftsX*; an isogenic derivative of CW1	This study
F. nucleatum CW2c	CW2 containing $pFtsX_{Flag}$	This study
F. nucleatum CW3	Δ*envC*; an isogenic derivative of CW1	This study
F. nucleatum CW3c	CW3 containing $pEnvC_{FLAG}$	This study
F. nucleatum CW4	Δ*radD*; an isogenic derivative of CW1	This study
F. nucleatum CW5	Δ*fadA;* an isogenic derivative of CW1	This study
A. oris MG1	Type strain	This study
S. oralis 34	RPS positive	(3)
Plasmid		
pHS30	*E. coli*/*Fusobacterium* shuttle vector, chloramphenicol /thiamphenicol resistance; cm^R/$thia^R$	(1)
pCWU5	Derivative of pUC19, cm^R	This study
pCWU7	Derivative of pCWU5, expressing *galK* under the control of the FN1529 promoter	This study
pCWU8	Derivative of pCWU7, Kan^R	This study
pCWU6	Derivative of pHS30	This study
pCK-galK	*Clostridium perfringens* vector expressing *galK*	(2)
$pFtsX_{FLAG}$	pCWU6 expressing H6-3XFLAG-FtsX	This study
$pFtsX_{FLAG}$(ΔECD1)	Derivative of $pFtsX_{FLAG}$ expressing FtsX without the ECD1 domain (residues 62-140)	This study
$pFtsX_{FLAG}$(ΔECD2)	Derivative of $pFtsX_{FLAG}$ expressing FtsX without the ECD2 domain (residues 241-250)	This study
$pFtsX_{FLAG}$(ΔD/E)	Derivative of $pFtsX_{FLAG}$ expressing FtsX without the E/D rich domain (residues 278-308)	This study
$pEnvC_{FLAG}$	pCWU6 expressing H6-3XFLAG-EnvC	This study
pCWU5-Δ*galK*	Derivative of pCWU5, deletion vector of *galK*	This study
pCWU8-Δ*ftsX*	Derivative of pCWU8, deletion vector of *ftsX*	This study
pCWU8-Δ*envC*	Derivative of pCWU8, deletion vector of *envC*	This study
pMinC	Derivative of pCWU8 expressing minC under the control of the *rpsJ* promoter	This study
