# Supplementary material for: Forward Genetic Dissection of Biofilm Development by Fusobacterium nucleatum: Novel Functions of Cell Division Proteins FtsX and EnvC
Source: mBio. 2018 Apr 24;9(2):e00360-18. doi: 10.1128/mBio.00360-18 (PMC5915739; doi:10.1128/mBio.00360-18)
Supplement: TABLE S3 [file mbo002183846st3.pdf]

Table S3: Primers used in this study

Primer	Sequence[(a)]	Used for
galK-up-F	GGCGGAATTCACAATATTAATGATTTAAAATAG	pCWU5-Δgalk
galK-up-R	GGCGGGTACCCCAATTAAATTCACTCTACCTGGTGA	pCWU5-Δgalk
galK-dn-F	GGCGGGTACCGCTAGTTTCTATATAGCAAATATTGGAG	pCWU5-Δgalk
galK-dn-R	GGCGTCTAGAAACAGCATCAATATGTCCTGTGATTAAG	pCWU5-Δgalk
catP-HS30-F	GGCGAACGTTAGTTATGGAAATAAGACTTAGAAG	pCWU5
catP-HS30-R	GGCGAACGTTTTAACTATTTATCAATTCCTGCA	pCWU5
Kan215-F	GGCGCATATGCCAAGCTAGCTTCACGCTGCCGCAAG	pCWU8
Kan215-R	GGCGCATATGCCGCTCAGAAGAACTCGTCAAGAAG	pCWU8
P1529-F	GGCGGTCGACAAAGTTGGATAAATTTAATTTAACTT	pCWU7
P1529-R	GGCGGCTAGCAAATTCAACCTCCCCTTTTTTATATT	pCWU7
galK-F	GGCGGCTAGCATGTTAGAAA ATTTAATAAAAGACTT	pCWU7
galK-R	GGCGAAGCTTTTATTTCACCTTTCCTGCTCCATCTC	pCWU7
rpsJcory-F	GGCGGAGCTCGCTGCAGAGGTACCAGTCGACACTTAG CAATGGTGTTGCAATGTC	pCWU6
rpsJcory-R	GGCGCATATGTTATTTATTGCACCTCCTTTAAATTAAC	pCWU6
mCherry-F	GGCGCATATGGTGAGCAAGGGCGAGGAGGAT	pCWU6
mCherry-R	GGCGAAGCTTGAGATCTGCTCGAGAGGATCCCTACTAC TTGTACAGCTCGTCCATG	pCWU6
PcatP-F	GGCGGAGCTCAACGAGTGAAAAAGTACTGGTCCCTAG	pFtsX$_{FLAG}$
PcatP-R	GGCGGGTACCGTTCCCTCTCAAATTCAAGTTTATCG	pFtsX$_{FLAG}$
Com-FtsX-F	GGCGGGTACCATGGAAGAAGATAAAATCTTACATGGAA TTCT	pFtsX$_{FLAG}$
Com-FtsX-R	AAACTCGAGTTACTTATCATCATCATCCTTATAATCTATA TCATGATCCTTATAATCTCCATCATGATCCTTATAATCAT GATGATGATGATGATGATCTTCATCATCTTCAAACT CAT	FtsX$_{FLAG}$
FtsX-F2	GGCGGGTACCTAAATTTTGAAAAAATGCTAGCATG	pEnvC$_{FLAG}$
com-EnvC-R	GGCGCTCGAGTTATTACTTATCATCATCATCCTTATAAT CTATATCATGATCCTTATAATCTCCATCATGATCCTTAAA TCATGATGATGATGATGATGAAATGTTGGTATTGGGTCA ATAGGTCTTAA	pEnvC$_{FLAG}$
RE-envC-F	GTATCTCATGCAAATTTTAATTCATCAATG	pEnvC$_{FLAG}$
RE-envC-R	TCTTCTTTTTAATCTATTTATATAG	pEnvC$_{FLAG}$
ftsX-up-F	GGCGGAGCTCTCAGCATTCGCAGCAAATGATGCAGCA	pCWU8-ftsX
ftsX-up-R	GGCGGGTACCTGAAAATATTTAATGCTACAACTGTAAT	pCWU8-ftsX
ftsX-dn-F	GGCGGGTACCCAGCAAATGTTGGAAGAATTGAATATG	pCWU8-ftsX
ftsX-dn-R	GGCGGTCGACCTAATTTCCTTTTTTCAGCTTCTATTTTC	pCWU8-ftsX
Re-ECD1-F	TAAATCTGCAACTATAAAAGAATTAA	pFtsX$_{FLAG}$(ΔECD1)
RE-ECD1-R	GAAGTGTATAAAGATGAATCATATTTAAAG	pFtsX$_{FLAG}$(ΔECD1)
Re-ECD2-F	ATTTGCATGAGATACATGTTTTCT	pFtsX$_{FLAG}$(ΔECD2)
Re-ECD2-R	ATTATTTTATGGCATTTTG	pFtsX$_{FLAG}$(ΔECD2)
Re-D/E-F	TTCAATTCTTCCAACATTTGCTGG	pFtsX$_{FLAG}$(ΔD/E)

Re-D/E-R	TAAGACAATTTTAGTATTTTTTCTTTTATCAG	pFtsX$_{FLAG}$(ΔD/E)
envC-up-F	GGCGGAGCTCATGAAAAAATTTTTATTATT AGCAGTAT	pCWU8-envC
envC-up-R	GGCGGGTACCTCTAATTTAG AAGTTTCTGT ATCTATTG	pCWU8-envC
envC-dn-F	GGCGGGTACCTTTATGCTGA TAAATTCCAA GGCTTGG	pCWU8-envC
envC-dn-R	GGCGGTCGACCTTTCAATAT TCTCTATTTTAGAGTCAAT	pCWU8-envC
ppnk-up-F	GGCGGGATCCTTTAGAAA ACATGTATCTCATGCAAATT	pCWU8-ppnk
ppnk-up-R	GGCGGGTACCTCCGCCTATAACAACTATATATTCAG	pCWU8-ppnk
ppnk-dn-F	GGCGGGTACCAAGATGAAGTAGAAATTTCTTACTCAAA	pCWU8-ppnk
ppnk-dn-R	GGCGAAGCTTTTATACCTCATTTAACATTTTATTTGCT	pCWU8-ppnk
amiD3-upF	GGCGGGTACCCTTGCTAAAGAAATATTATTTTTAAAG	pCWU8-amiD3
amiD3-upR	GGCGGGTACCAACACTATCTTTTGATTTATCTATTATT	pCWU8-amiD3
amiD3-dnF	GGCGGGTACCATATTGCAGATAGATTAGAGATGAG	pCWU8-amiD3
amiD3-dnR	GGCGAAGCTTTTCCAGAAGCTTCTGTCTTTGGAACTCT	pCWU8-amiD3
rpsJ-F	GGCGGAGCTCGTTTTTTCTAAGTCAATTTCAAATTCAG	pMinC
rpsJR	AAAAGGTACCTGCACCTCCTTTAAATTAACTACAT	pMinC
minC-F	GGCGGGTACCGGGAGGTAAATATGAGCAACCATGTAA TAATAAAAGG	pMinC
minC-R	GGCGCTCGAGTTATATCAACTCCTCAACAACTATTTCTT	pMinC
RT-ftsX-F	AGCAATCTAAGGAACAAGGTTT	RT-PCR *ftsX*
RT-ftsX-R	TCAAGTCCTGTATTGGCATTAT	RT-PCR *ftsX*
RT-16s-F	GGTTAAGTCCCGCAACGA	RT-PCR *16s*
RT-16s-R	CATCCCCACCTTCCTCCTAC	RT-PCR *16s*

[a] Underlined are the restriction sites in the primers.
